# *In silico* and *in vitro* study of bioactive compounds of *Nigella sativa* for targeting neuropilins in breast cancer

**DOI:** 10.3389/fchem.2023.1273149

**Published:** 2023-10-11

**Authors:** Imran Zafar, Arfa Safder, Hassan Imran Afridi, Sania Riaz, Rizwan -ur-Rehman, Ahsanullah Unar, Fakhar Un Nisa, Abdel-Rhman Z. Gaafar, Mohammed Bourhia, Gezahign Fentahun Wondmie, Rohit Sharma, Dileep Kumar

**Affiliations:** ^1^ Department of Bioinformatics and Computational Biology, Virtual University Pakistan, Lahore, Pakistan; ^2^ Institute of Molecular Biology and Biotechnology, The University of Lahore, Punjab, Pakistan; ^3^ National Center of Excellence in Analytical Chemistry, University of Sindh, Jamshoro, Pakistan; ^4^ Faculty of Health and Life Sciences, Department of Bioinformatics and Biosciences, Capital University of Science and Technology, Islamabad, Pakistan; ^5^ Department of Precision Medicine, University of Campania “L. Vanvitelli”, Naples, Italy; ^6^ Depatment of Molecular Biology, Virtual University of Pakistan, Lahore, Pakistan; ^7^ Department of Botany and Microbiology, College of Science, King Saud University, Riyadh, Saudi Arabia; ^8^ Department of Chemistry and Biochemistry, Faculty of Medicine and Pharmacy, Ibn Zohr University, Laayoune, Morocco; ^9^ Department of Biology, Bahir Dar University, Bahir Dar, Ethiopia; ^10^ Department of Rasa Shastra and Bhaishajya Kalpana, Faculty of Ayurveda, Institute of Medical Science, Banaras Hindu University, Varanasi, India; ^11^ UC Davis Comprehensive Cancer Center, University of California, Davis, Davis, CA, United States; ^12^ Poona College of Pharmacy, Bharati Vidyapeeth (Deemed to be) University, Pune, India; ^13^ Centre for Advanced Research in Pharmaceutical Sciences, Poona College of Pharmacy, Pune, India

**Keywords:** *Nigella sativa*, breast cancer, neuropilins, oncogenes, phytochemical screenings, bioactive compounds, molecular docking, molecular dynamic simulation

## Abstract

**Introduction:** Breast cancer poses a significant global challenge, prompting researchers to explore novel approaches for potential treatments.

**Material and Methods:** For *in vitro* study we used thin layer chromatography (TAC) for phytochemical screening, total antioxidant capacity (TLC) assay for antioxidant capacity, and hemolytic activity test for toxicity of Neuropilins (NRPs). We performed bioinformatic analyses to predict protein structures, molecular docking, pharmacophore modeling, and virtual screening to reveal interactions with oncogenes. We conducted 200 ns Molecular Dynamics (MD) simulations and MMGBSA calculations to assess the complex dynamics and stability.

**Results:** We identified phytochemical constituents in *Nigella sativa* leaves, including tannins, saponins, steroids, and cardiac glycosides, while phlobatannins and terpenoids were absent. The leaves contained 9.4% ± 0.04% alkaloids and 1.9% ± 0.05% saponins. Methanol extract exhibited the highest yield and antioxidant capacity, with Total Flavonoid Content at 127.51 ± 0.76 mg/100 g and Total Phenolic Content at 134.39 ± 0.589 mg GAE/100 g. Hemolysis testing showed varying degrees of hemolysis for different extracts. In-silico analysis indicated stable Neuropilin complexes with key signaling pathways relevant for anti-cancer therapy. Molecular docking scores at different possesses (0, C-50, C −80, C-120,C −150, C −200 ns) revealed strong hydrogen bonding in the complexes and showed −12.9, −11.6, and −11.2 binding Affinities (kcal/mol) to support their stability. Our MD simulations analysis at 200ns confirmed the stability of Neuropilin complexes with the signaling pathways protein PI3K. The calculated binding free energies using MMGBSA provided valuable quantitative information on ligand potency on different time steps. These findings highlight the potential health benefits of *N. sativa* leaves and their possible role in anti-cancer treatments targeting angiogenesis.

**Conclusion:** *Nigella sativa* leaves have shown significant medical potential due to their bioactive compounds, which exhibit strong properties in supporting organogenic processes related to cancer. Furthermore, studies have highlighted the promising role of neuropilins in anticancer treatment, demonstrating stable interactions and potential as targeted therapy specifically for breast cancer.

## 1 Introduction

Breast cancer remains a complex and multifaceted condition that represents a major global threat to the health of millions of women (Tan et al., 2021; Ahmad et al., 2022). Despite advances in treatment, innovative therapeutic techniques are constantly being sought to address its complexity. One promising area of research is neuropilins (NRPs), transmembrane receptors that play crucial roles in breast cancer biology and various signaling pathways (Islam et al., 2022). Targeting national reforms as therapeutic options has great potential due to their multifunctional nature and their involvement in cancer-related processes. Traditionally, cancer therapy has focused on specific oncogenes or pathways, but recent studies have highlighted the intricate crosstalk between multiple oncogenes known as multi-coded oncogenes (Pham, 2022). These multi-encoded oncogenes present exciting opportunities for precision medicine, as they influence treatment resistance, disease progression, and cancer heterogeneity. Understanding how genes and proteins work in breast cancer will shed light on the underlying molecular processes that drive the disease. Ongoing research and clinical exploration in this area involves the use of inhibitors or immunotherapies to target specific molecules. As our knowledge base expands, these molecular targets offer great potential to advance individualized breast cancer therapy and improve patient outcomes.

CDK4 (cyclin-dependent kinase 4) is essential for cell division and works with cyclin D1 to drive the cell cycle from the G1 phase to the S phase, allowing cells to proliferate. In some subtypes of breast cancer (Ding et al., 2020), overexpression or amplification of CDK4 leads to uncontrolled cell proliferation and tumorigenesis. EGFR (epidermal growth factor receptor), a receptor tyrosine kinase, plays an important role in cell growth, proliferation, and survival (Tripathi et al., 2020). Abnormal EGFR expression or mutations in breast cancer are associated with aggressive tumor behavior and resistance to treatment. Targeting of EGFR with specific inhibitors has been investigated as a possible treatment strategy for certain subtypes of breast cancer (Mir et al., 2020). The small GTPases of the RAS family act as molecular switches in intracellular signaling, controlling cell growth and survival. Mutations in the RAS genes (e.g., KRAS, HRAS, NRAS) can cause abnormal signaling and uncontrolled cell proliferation, which are commonly found in other types of cancer but occasionally occur in breast tumors (Seetharaman Thillai Villalan, 2020). BRAF (B-Raf), a serine/threonine kinase in the MAPK signaling pathway, rarely shows activating mutations in breast cancer but is more common in other malignancies such as melanoma (Zaman et al., 2019). However, in certain breast cancer subtypes with BRAF mutations, targeting them may offer viable treatment options. The PTEN (Phosphatase and TENsin homolog) tumor suppressor gene regulates cell growth and survival by inhibiting the PI3K/AKT pathway (Jiang et al., 2020). Loss or inactivation of PTEN in a fraction of breast tumors is associated with aggressive tumor behavior. The PI3K/AKT pathway, which is influenced by PI3K, PIP3, and AKT, plays an important role in hormone receptor-positive and HER2-positive breast cancers, contributing to tumor progression and treatment resistance (Agostinetto et al., 2021).

In recent years, *Nigella sativa* has attracted increasing attention as a potential source of bioactive compounds with promising therapeutic properties. These compounds have been explored for their various pharmacological effects, including anti-inflammatory, antioxidant, and anticancer activities. However, there is a notable knowledge gap in understanding the precise molecular mechanisms underlying the therapeutic potential of *N. sativa* and its metabolites, particularly in the context of cancer therapy. This gap is particularly evident in the limited exploration of its interactions with key cellular components involved in cancer progression, such as neuropilins (NRPs) and their binding proteins. We aim to investigate how bioactive compounds from *N. sativa* interact with NRPs, uncovering their potential in cancer treatment.

Drug design techniques, such as molecular docking and molecular dynamic simulations, have been used to study DNA binding and stability in solvents (Ali et al., 2023c). The new most dynamic scoring, particularly with molecular mechanics/Poisson−Boltzmann surface area (MM/PBSA) and QM/MM (quantum mechanics/molecular mechanics), has reduced false positives in virtual screening (Parves et al., 2023). MM/GBSA-based drugs show promising activity, often superior to experimental therapies, leading to cost-effective and efficient high-throughput inhibitor screening (Tabti et al., 2023). Molecular dynamics can help restore unfolded proteins to their physiologically folded state, helping to discover new therapies (Chalbatani et al., 2022). Virtual sensing and docking provide insights into ligand orientation at the active site, while molecular dynamics modeling tracks stable bond formation and molecular mechanisms (Saher Javaid, 2023). In-depth molecular dynamic (MD) simulationstudies have been carried out to understand the polymer dynamics and pharmacological point of view, which contributes to the drug development process.

Neuropilins have shown promise in the treatment of various types of cancer by reducing tumor growth, blocking angiogenesis, and potentially improving chemotherapy delivery (Liu et al., 2021). As a key therapeutic option, its unique mode of action and focus on survival outcomes make it valuable in the treatment of cancer (Chalbatani et al., 2022). However, careful evaluation and consideration of specific patient cases and ongoing research are essential for optimal results. Although neuropilins primarily function as vascular endothelial growth factor (VEGF) coreceptors in cancer, their interactions with CDK4, EGFR, RAS, BRAF, PI3K, and PTEN are not fully understood (Li et al., 2023). The complex interactions between VEGF/neuropilin signaling, tumor development, angiogenesis, and cell survival are topics of ongoing investigation in various types of cancer.

This ongoing research is focused on exploring the anticancer potential of organic molecules, specifically neuropilins, in the treatment of breast cancer. The study takes an integrated approach, combining *in vitro* and silico methods. Computational tools and bioinformatics are used to analyze interactions between neuropilins and specific oncogenes, identifyingnew compounds that can modify neuropilin activity and disrupt cancer signaling pathways. *In vitro* experiments involve cell culture studies to investigate how neuropilins impact the expression and activity of multi-encoded oncogenes, thereby validating the computational findings. Ultimately, the research aims to decipher the complex network of neuropilin-mediated interactions in breast cancer, offering the potential for personalized treatment strategies that could improve patient prognosis. This comprehensive study has the potential to advance our understanding of breast cancer biology and open up new avenues for targeted therapy against this devastating disease.

The scope of this research is broad, encompassing several key aspects. Firstly, it involves the identification and characterization of new compounds that can modify neuropilin activity, a critical step in disrupting cancer signaling pathways. Additionally, the study employs computational tools and bioinformatics to analyze the intricate interactions between neuropilins and specific oncogenes, providing valuable insights into potential mechanisms of action. These computational findings are further validated through *in vitro* experiments using cell culture studies, where the effects of neuropilins on the expression and activity of oncogenes are examined. Beyond the laboratory work, the research seeks to unravel the intricate network of neuropilin-mediated interactions in breast cancer, offering the promise of personalized treatment strategies that could enhance patient outcomes. This comprehensive approach not only advances our knowledge of breast cancer biology but also holds the potential to revolutionize targeted therapies for this devastating disease.

## 2 Materials and methods

In this study, we embarked on a comprehensive exploration of the bioactive compounds found in *N. sativa* to target neuropilins in the context of breast cancer.

**FIGURE 1:**
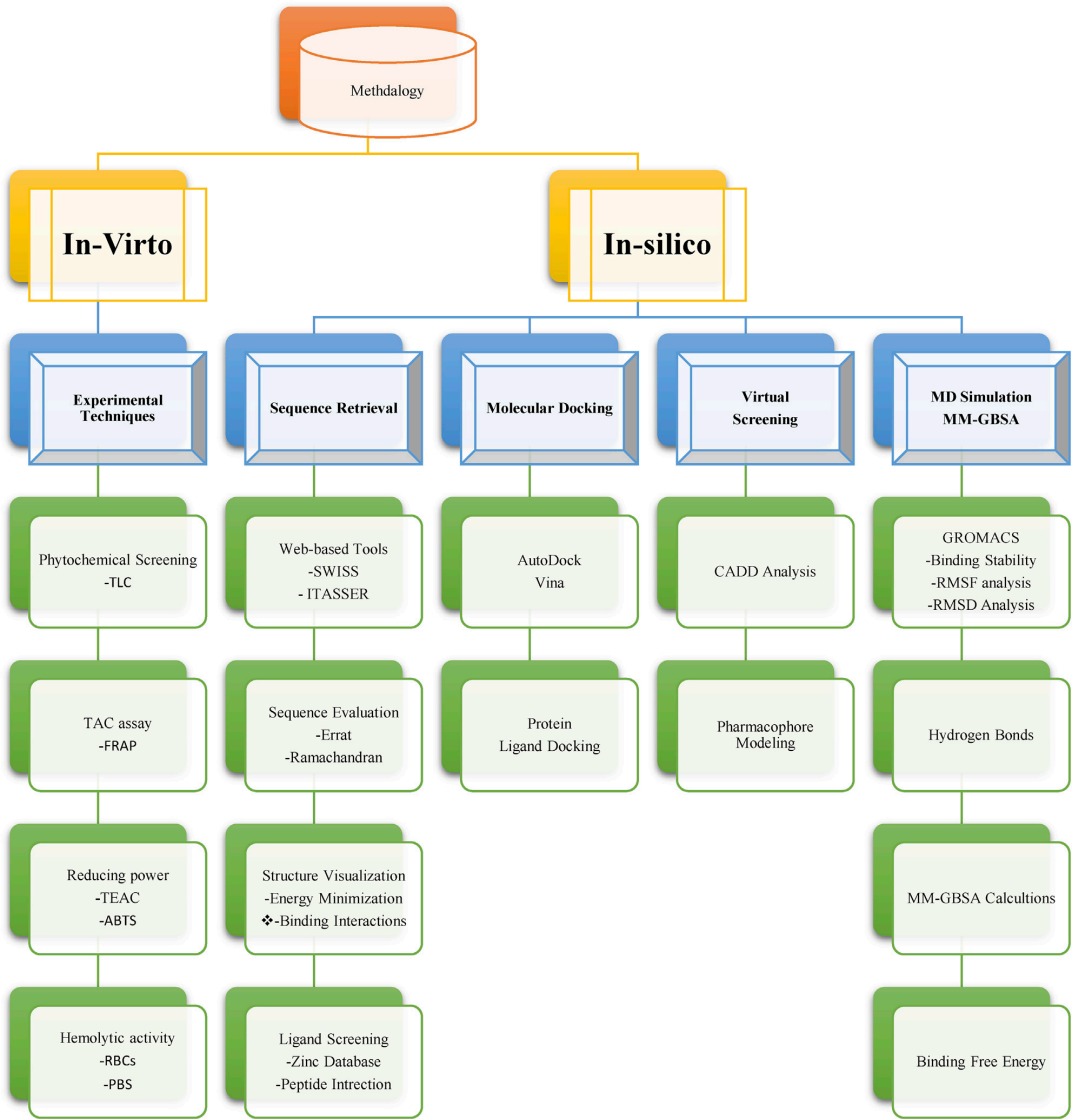
A flowchart of appliedmethodology for targeting neuropilins in breast cancer with *Nigella sativa* compounds.

Our investigative journey commenced with the careful selection of *N. sativa* compounds, followed by the formation of an intricate experimental setup for *in vitro* analysis. Simultaneously, we employed cutting-edge computational techniques for *in silico* evaluation. These parallel approaches synergistically unravel the potential of *N. sativa* in targeting breast cancer. Through a systematic and integrated methodology as mentioned in Figure 1, aspire to contribute valuable insights towards the development of novel therapeutic strategies in breast cancer treatment.

### 2.1 Data sets

Seeds of *Nigella sativa* were carefully collected at random, thoroughly washed, and dried at room temperature to avoid contamination. To extract the desired components, a 50-g powder was obtained and macerated with methanol for 7 days. The active substances were then recovered in the resulting aqueous filtrate. After the evaporation of the methanol, a solid extract was obtained that allowed precise measurements. For the docking analysis to ensure the best conformation, the 3D structure of the 2I3B target protein (PDB ID: 2I3B) was generated using Swiss PDB Viewer (https://spdbv.unil.ch/). Next, a variety of receptors, including 2I3B, 3G5X, 6T51, 4R5Y, 2N7X, and 6CU6, were tested with Neuropilins ligand in docking assays. Protein-ligand interactions and binding energies were analyzed using the AutoDock Vina program, which also provided valuable information on the strength of binding between receptors and ligands. To identify proteins critical for drug absorption, distribution, metabolism, and excretion, the study’s selected proteins were obtained from the Swiss target prediction site ADME (http://www.swissadme.ch/). Furthermore, the protein structures required for the docking simulations were retrieved from the Protein Data Bank (PDB) database (https://www.rcsb.org/).

### 2.2 Experimental techniques

#### 2.2.1 Phytochemical screening

We used thin-layer chromatography (TLC) to identify important bioactive substances in *N. sativa* seeds, such as tannins, phlorotannins, saponins, steroids, and terpenoids (Li et al., 2021). First, we extracted these phytochemicals from powdered *N. sativa* seeds with ethanol or methanol. We then plated the extracted solution on a silica gel-coated TLC plate. Capillary action allowed the solvent to rise the plate and transport the phytochemicals. The TLC plate was then developed in a mobile phase solvent chamber. After proper development, we examined the TLC plate using ultraviolet light and other techniques. By comparing the spots with the standard references and calculating the Rf values, we determined the presence or absence of these compounds in the *N. sativa* seed extract. TLC provided valuable qualitative data on the chemical composition of the seeds, which revealed their potential health-promoting properties (Engel and Schiller, 2021).

#### 2.2.2 TAC assay

In this study, we used the Total Antioxidant Capacity (TAC) test to assess the remarkable antioxidant potential of *N. sativa* seeds (Zouirech et al., 2022; Fatima et al., 2023). The TAC test is a valuable technique to assess the cumulative antioxidant activity of natural compounds. To do this, we used the FRAP test (Rachmayanti et al., 2023), which measures the ability of antioxidants to convert an iron-tripyridyltriazine (Fe3+-TPTZ) complex to its ferrous (Fe2^+^) form, leading to the formation of a distinctive blue-colored complex. To obtain the extract, we used an ultrasonic device to finely grind the *N. sativa* seeds and then extracted them with methanol. After centrifugation, the supernatant containing the extract was obtained. Next, we prepared several standard solutions with FeSO4 to establish a calibration curve, which allowed us to accurately calculate the antioxidant capacity. We mixed a constant amount of the FRAP reagent with varying amounts of the *N. sativa* seed extract in separate test tubes. After incubating the reaction mixes for 30 min, we measured the absorbance at the appropriate wavelength. Using the calibration curve, we accurately calculated the FRAP values of the *N. sativa* seed extract, thus estimating its total antioxidant capacity in terms of FeSO4 equivalents (mol FeSO4/g extract) or any other suitable unit. Our data were carefully presented as means with corresponding standard deviations (SD). Notably, each experiment was performed in triplicate to ensure robustness and reliability.

#### 2.2.3 Reducing power determination

In this study, we used the Trolox equivalent antioxidant capacity (TEAC) test as a robust method to assess the reducing power and antioxidant activity of *N. sativa* leaves (Elveny et al., 2021). To achieve this, different solvent extracts of the leaves of the plant were prepared at different concentrations. The TEAC test involved the generation of the reactive towards most antioxidants (ABTS) radical cation by combining ABTS and potassium persulfate in water (Ilyasov et al., 2020). Then, each plant extract was mixed with ABTS radical cationic solution and sodium phosphate buffer and allowed to incubate in the dark for 6 min at room temperature. With a spectrophotometer, we measured the absorbance at 734 nm after incubation, which allowed us to evaluate the reducing power. To establish a standard curve, we used Trolox, a water-soluble vitamin E mimetic, in different concentrations. By calculating the Trolox TEAC for each extract concentration, we were able to determine the antioxidant potential of the plant sample. By comparing the TEAC values to the Trolox standard curve, we were able to further measure the efficiency of the plant in scavenging free radicals and its potential as a source of valuable antioxidants.

#### 2.2.4 Hemolytic activity

In this study, we developed an innovative approach to assess the hemolytic activity of *N. sativa* leaves, building on existing research in the field (Greco et al., 2020). To ensure the integrity of our blood samples, fresh human blood (3 mL) was carefully collected into heparinized tubes and then gently centrifuged at 850 g for 5 min to prevent clotting. After decanting the supernatant, the isolated red blood cells (RBCs) were subjected to three washes with sterile isotonic phosphate-buffered saline (PBS) (5 mL, pH 7.4) in a refrigerated environment. The washed red blood cells were then concentrated to 7.068 × 10 8 cells per ml and suspended in chilled PBS (20 mL). For sample preparation, 2 mL Eppendorf tubes were used. To facilitate the interaction between the extract of the *N. sativa* leaf plant and red blood cells, 20 μL of the extract was added to each Eppendorf tube, followed by 180 μL of the blood cell suspension. The samples were then incubated at 37°C for 35 min. After this incubation period, the tubes were briefly placed on ice for 5 min to stop the reaction and then centrifuged at 1,310 g for 5 min. To stabilize the samples for analysis, 100 μL of the supernatant was carefully removed from each Eppendorf tube and further diluted with chilled PBS (900 μL). Then, the diluted supernatant from each tube was transferred in 200 μL portions to the wells of a 96-well plate. To establish baseline controls, 0.1% Triton X-100 (a positive control) and PBS (a negative control) were added to separate wells of the 96-well plate. Using a precision spectrophotometer (Koetsier and Cantor, 2019), we measured the absorbance of each well at 576 nm. Using the formula % hemolysis = (absorbance of sample/absorbance of control) × 100, we calculate the percentage of hemolysis by comparing the absorbance of the sample to that of the control.

### 2.3 Bioinformatic analysis

#### 2.3.1 Structure prediction

In this rigorous investigation, we acquired the FASTA-formatted amino acid sequences of vital proteins, namely CDK4, EGFR, RAS, BRAF, PTEN, and PI3K, from UniProt (Zafar et al., 2022; Ali et al., 2023b). To determine suitable templates for these target proteins, a comprehensive Protein Data Bank search was performed using BLASTp as per the method of (Mahram and Herbordt, 2015; Dana et al., 2020). Using the model SWISS and ITASSER, we generated predicted 3D structures, which underwent careful cross-validation (Kiefer et al., 2009). Quality assessment of these structures was carefully performed using online validation tools such as ERRAT, Verify3D, and Rampage (Dym et al., 2012). Chimera managed to improve the 3D structures of some chemical substances that interact with proteins. In addition, a complete PSSE (Protein Secondary Structural Elements) survey was simulated. This comprehensive approach not only led to the identification of promising anticancer phytocompounds, but also facilitated the accurate prediction and validation of the 3D structures of TCDK4, EGFR, RAS, BRAF, PTEN, and PI3K.

#### 2.3.2 Molecular docking

In this work, the structure of the DNA duplex receptor was docked from the Protein Data Bank using Autodock Vina 4.2 as per the method of earlier researchers (Rauf et al., 2015; Dana et al., 2020). The aim was to investigate the molecular relationships between neuropilins and the proteins CDK4, EGFR, RAS, BRAF, PTEN, and PI3K. Based on the method of Al-Sha’er et al. (2015) procedure involved curation of missing side chain residues, determination of the binding pocket of neuropilins using X-ray co-crystallized structures, calculation of residue positions, energy minimization studies, and docking using Lamarck’s genetic algorithm.

#### 2.3.3 Molecular dynamic simulations

We conducted molecular dynamics (MD) simulations on six protein-ligand complexes (2I3B, 3G5X, 6T51, 4R5Y, 2N7X, and 6CU6) using GROMACS 2020.2. The topologies and configuration files for both proteins and ligands were generated using CHARMM-GUI and the ParamChem servers, following the methodology of previous researchers (Jo et al., 2013; Dana et al., 2020). The systems were solvated in TIP3P water, and Na+ and Cl-ions were added for charge neutrality. Periodic boundary conditions (PBC) were applied, with a nonbonded interaction cutoff distance of 12 Å. CHARMM36 force fields were utilized for building the complexes (Jo et al., 2014). The system was energy-minimized followed by NVT and NPT after 125 ps equilibration and runs at 300.15 K, where 200 ns production MD run in the NPT conditions. H-bonds were constrained by experimental and computational methods network-based cellular signatures (LINCS). The temperature was set to 300 K by the V-scaling thermostat 28 with a time coupling constant of 0.1 ps. Simulations revealed the kinetics of Protein–ligand (un) binding and stability interaction throughout biomolecular processes.

#### 2.3.4 Trajectory analysis

GROMACS techniques were used to evaluate MD simulations (Rawat et al., 2021). Gmx-rms was used to calculate the root-mean-square deviation (RMSD) of the ligand and protein atom sites, and Gmx-RMSD was used to calculate the RMSD based on the C-alpha atoms of the protein. Using gmx-hbond, hydrogen bonding at the protein-ligand interface was calculated. Using gmx-gyrate, the radius of gyration of each protein atom was determined. gmx-distance was used to calculate the distance between the center of mass of the simulation between the protein and the ligand. Frequency analysis and visualization of the trajectory of protein-ligand interactions were performed using visual molecular dynamics (VMD) software. These investigations provide important information about the dynamics, interactions, and stability of the protein-ligand complex.

#### 2.3.5 MM-GBSA calculassions

The Molecular Mechanics combined with the Generalized Born Surface Area (MM-GBSA) were used to compute the binding free energies of the ligand-protein complexes. The MM-GBSA free binding energy was determined via the Python script thermal mmgbsa.py in the simulation trajectory with the optimized potentials for liquid simulations (OPLS-2005) force field after every 10ns. The binding free energy of Prime MM-GBSA (kcal/mol) was estimated using the principle of additive to obtain the equation of ΔG binding calculations

$${∆G}_{bind}={\Delta E}_{MM}+{\Delta G}_{Solv}+{\Delta G}_{SA}$$

Where ΔG_bind_ designates the binding free energy, ΔE_MM_ the difference between the ligand-protein complex’s free energies and the total energies of the protein and the ligand in isolated form, ΔG_Solv_ the difference between the solvation energies of the ligand-receptor complex and the unbound state solvation energies sums and ΔG_SA_ designates the surface area for both protein and ligand differences.

## 3 Results

### 3.1 Phytochemical screening of *Nigella sativa* seeds

The thin layer chromatography (TLC) results of the phytochemical analysis of *N. sativa* seeds indicated the presence of some chemical compounds and revealed the absence of others. TLC analysis verified that the *N. sativa* seed extract included tannins, saponins, steroids, and cardiac glycosides. However, no phlobatanins or terpenoids were found in the sample.

TLC is an important qualitative technique that made it possible to identify these phytochemicals based on their unique Rf values. The presence of tannins (Rf = 0.35), saponins (Rf = 0.80), steroids (Rf = 0.43), and cardiac glycosides (Rf = 62) in the extract was verified by the appearance of appropriate points on the TLC plate in selected Rf hosts. On the other hand, the lack of spots that would have indicated the presence of phlorotannins and terpenoids in the *N. sativa* seed extract suggested that they were not present. Several therapeutically useful plant components were discovered in the present investigation of *N. sativa*, as listed in Table 1. The plant included cardiac glycosides, alkaloids, tannins, and saponins, but no steroids or terpenoids.

**TABLE 1:** Qualitative analysis of the phytochemicals of the *Nigella sativa* leaves extracts.

Plant	Alkaloid	Tannin	Saponin	Steroid	Phlobatinin	Terpenoid	Cardiac glycoside
*Nigella sativa*	+	+	+	-	+	-	+

This study evaluated qualitative criteria to distinguish closely related variations with similar pharmacological activity. It also quantitatively analyzed the chemical content of *Nigella sativa* leaves, as presented in the data provided in Table 2. The results support the health benefits of *N. sativa* seeds, with phytochemicals identified as having natural antibiotic properties, such as saponins and tannins. The presence of cardiac glycosides suggests a potential role in the treatment of moderate to severe myocardial infarctions. Quantitative estimation of the crude chemical components of the *N. sativa* leaf extract revealed a significant content of alkaloids (9.40%) with potential pharmacological effects. Notable alkaloids found in *N. sativa* include piperine, nigellain, and afilin. In addition, there are saponins (1.90%), known for their various biological functions and the ability to form stable foams when mixed with water. The plant contains saponins such as cardamom, quercetin, and kaempferol.

**TABLE 2:** Quantitative estimation of the percentage of crude chemical constituents in *Nigella sativa* leaves extracts.

Chemical constituents of *Nigella sativa*	Percentage (%) of crude chemical constituent
Alkaloids	9.4 ± 0.04
Saponin	1.9 ± 0.05

### 3.2 Percentage yield of plant extracts

**FIGURE 2:**
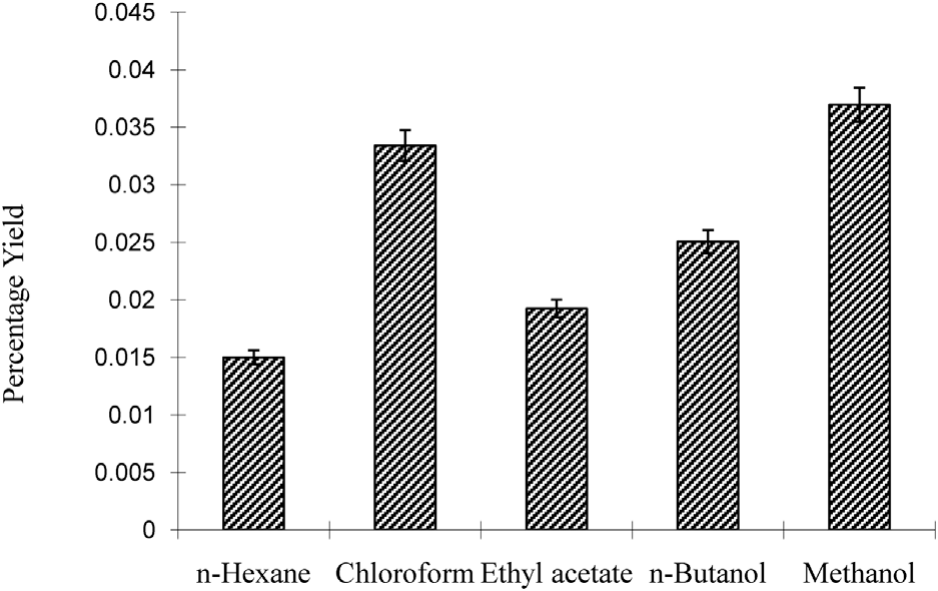
Percentage yield of *Nigella sativa* leaves extracts.

The results of the investigation indicate that the choice of solvent significantly affected the yield percentage of the plant extracts. N-hexane, which is nonpolar, gave the lowest yield (0.015), while chloroform, a moderately polar solvent, gave the highest yield (0.035). Butanol (0.025) and acetone (0.025) showed moderate yields, making them suitable for polar and nonpolar molecules, respectively. In particular, methanol showed the highest yield (0.035) and was found to be effective in extracting a variety of phytochemicals, as shown in Figure 2.

#### 3.2.1 Total flavonoid content

The concentrations of total flavonoids (TFC) in dry extracts of *Nigella sativa* leaves extracted with different solvents are shown in Table 3. The maximum TFC was found in methanol extract (127.51 ± 0.76 mg/100 g), while the lowest was found in n-hexane. (4.47 0.05 mg/100 g). The results show that methanol is the best solvent for flavonoid extraction, while n-hexane is the worst. Many beneficial biological properties of flavonoids. This information can be used by researchers and producers for future applications.

**TABLE 3:** Optimal flavonoid extraction from *Nigella sativa* leaves.

Extract solvent	Total flavonoid contents (CE mg/100 g) in dry leaves extracts	Standard deviation	Confidence interval (95%)	Flavonoid potential (relative to methanol extract) (%)
n-Hexane	4.47	±0.05	[4.42, 4.52]	3.50
Chloroform	32.17	±0.52	[31.65, 32.69]	25.24
Ethyl acetate	17.48	±0.17	[17.31, 17.65]	13.71
n-Butanol	25.98	±0.50	[25.48, 26.48]	20.38
Methanol	127.51	±0.76	[126.75, 128.27]	100

CE, catechin equivalent.

#### 3.2.2 Total phenolics content

The totall phenolic content (TPC) of *Nigella sativa* leaves was measured in milligrams of gallic acid equivalents (GAE) per 100 g of dry weight during the study examination of plant leaf extracts made with different solvents. Data as mentioned in Table 4, the methanol extract had the highest TPC (134, 390, 589 mg GAE/100 g) and the n-hexane had the lowest (23, 250, 102 mg GAE/100 g). The ability of phenols to improve the nutritional value and quality of foods is well documented. The highest TPC was obtained by methanol extraction, which has potential applications in the food industry.

**TABLE 4:** Total phenolic contents in *Nigella sativa* leaves extracts.

Extract solvent	Total phenolic contents (GAE mg/100 g) in dry leaves extracts	Standard deviation	Confidence interval (95%)	Antioxidant potential (relative to methanol extract) (%)
n-Hexane	23.25	±0.102	[23.003, 23.497]	17.32
Chloroform	111.55	±0.136	[111.282, 111.818]	83.07
Ethyl acetate	26.95	±0.056	[26.894, 27.006]	20.06
n-Butanol	125.09	±0.101	[124.989, 125.191]	93.14
Methanol	134.39	±0.589	[133.814, 134.966]	100

GAE, gallic acid equivalent.

### 3.4 Inhibition of oxidation

To determine of antioxidant potential of *Nigella sativa* seed extract, we applied the total antioxidant potential (TAC) test based on the iron-reducing antioxidant power (FRAP) method. In terms of FeSO4 equivalents (mol FeSO4/g extract), the results are shown in Table 5.

**TABLE 5:** FRAP values of *Nigella sativa* seed extract.

Sample volume (µL)	FRAP value (µmol FeSO4/g extract)
20	15.23
40	27.41
60	34.89

**FIGURE 3:**
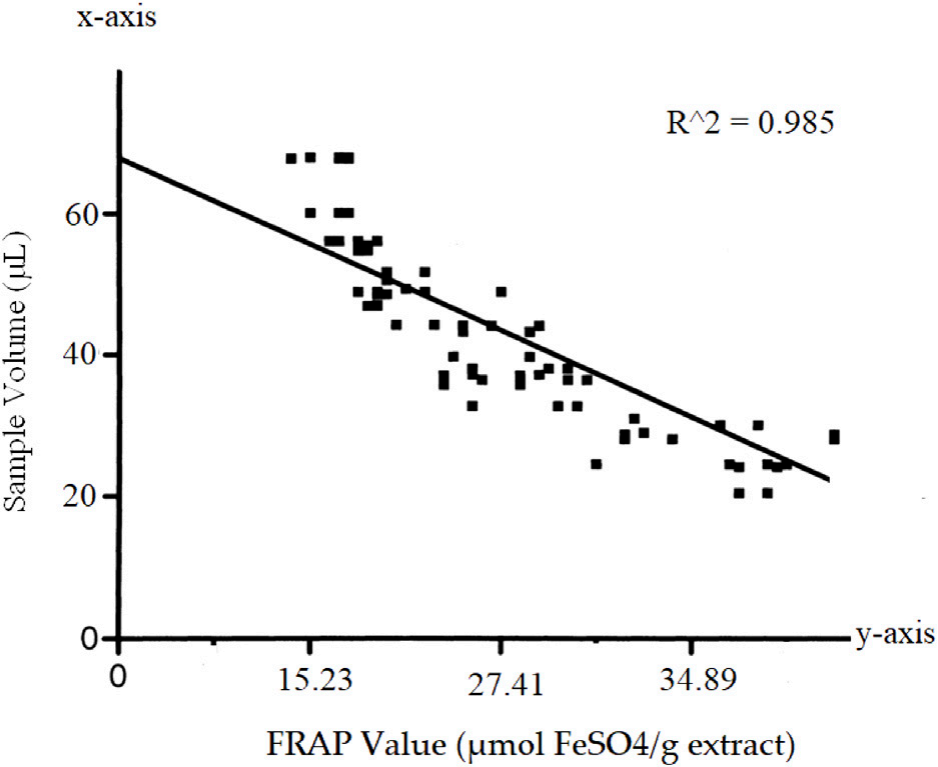
Calibration curve.

The FRAP values were calculated based on the calibration curve obtained using standard solutions of FeSO4. The calibration curve showed a linear relationship between the sample volume and the FRAP values (R^2 = 0.985) as in Figure 3 Scatterplot showing the calibration curve for the FeSO4 standard solutions. The x-axis represents the sample volume (µL), and the y-axis represents the corresponding FRAP values (µmol FeSO4/g extract).]

We observed a concentration-dependent relationship in the antioxidant capacity of the *Nigella sativa* seed extract. As we increased the sample volume, the FRAP values also increased, indicating a heightened antioxidant potential within the extract. The calculated FRAP values demonstrated the extract’s ability to reduce the Fe3+-TPTZ complex, emphasizing its potency as a strong antioxidant. The concentration-dependent response observed in the calibration curve further confirmed that the extract exhibits a cumulative antioxidant effect, with larger volumes leading to greater antioxidant activity.

### 3.5 Reducing power

In this study, we used the Trolox Equivalent Antioxidant Capacity (TEAC) assay to determine the reducing power of *N. sativa* leaves. Different solvent extracts from the leaves of the plants were prepared at concentrations between 2.5 and 1.0 mg/mL. Then, the extracts were sent to the TEAC assay to evaluate their antioxidant activity.

Data as mentioned in Table 6, the absorbance at 734 nm was measured for each extract concentration after incubation with the ABTS radical cation. The resulting TEAC values, which represent the reducing power of the extracts, were calculated based on the Trolox standard curve. The data indicate that as the concentration of the *Nigella sativa* leaf extracts decreased, the TEAC values also decreased, indicating a lower reducing power and antioxidant activity. On the contrary, higher extract concentrations showed stronger reducing power, as indicated by higher TEAC values.

**TABLE 6:** Reducing Power Determination using Trolox Equivalent Antioxidant Capacity (TEAC) Assay.

Sample concentration (mg/mL)	Absorbance at 734 nm	TEAC value
2.5	0.250	0.042
2.0	0.212	0.072
1.5	0.180	0.102
1.0	0.148	0.132

**FIGURE 4:**
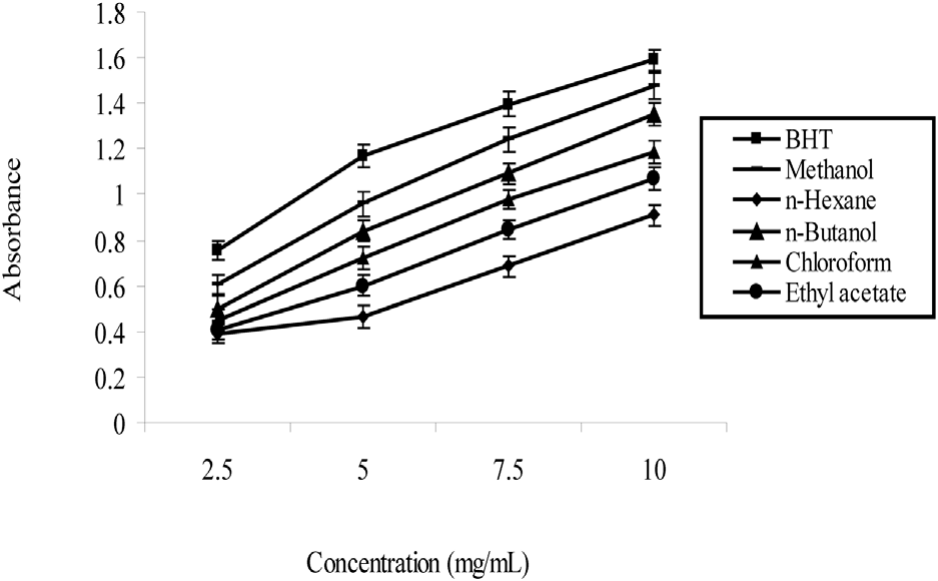
Trolox Standard Curve of *Nigella sativa* leaves extracts.

The Trolox standard curve was essential for the quantitative analysis, allowing us to convert the absorbance values of the plant extracts into TEAC values, providing a direct comparison with the antioxidant capacity of Trolox. The results shown in Figure 4 provide valuable information on the antioxidant potential of *N. sativa* leaves and demonstrate the usefulness of the TEAC assay as a reliable method to assess the reducing power of plant samples.

### 3.6 Hemolytic activity

For hemolytic activity, we used the negative control (PBS) and the positive control (0.1% Triton X-100). As expected, the negative control exhibits the lowest absorbance (0.082) and 0% hemolysis of the reference absorbance. The positive control, on the other hand, causes total hemolysis and shows a high absorbance (1.243) and 100% hemolysis as mentioned in Table 7.

**TABLE 7:** Hemolytic activity of different extracts from *Nigella sativa* leaves.

Sample	Absorbance at 576 nm	% Hemolysis
Negative Control (PBS)	0.082	0.0
Positive Control (0.1% Triton X-100)	1.243	100.0
Sample 1 (*Nigella sativa* extract A)	0.205	16.5
Sample 2 (*Nigella sativa* extract B)	0.078	3.7
Sample 3 (*Nigella sativa* extract C)	0.135	10.9
Sample 4 (*Nigella sativa* extract D)	0.098	7.9

Samples 1 to 4 are different extracts of *Nigella sativa* leaves that were tested for their hemolytic activity. Each sample shows a varying amount of absorbance, corresponding to different degrees of hemolytic activity. Such as sample 1 has an absorbance of 0.205 and a hemolysis of 16.5%, indicating a significant hemolytic effect. Sample 2, on the other hand, shows a lower absorbance of 0.078% and 3.7% hemolysis, indicating less hemolytic activity. With absorbance values of 0.136 and 0.098 for sample 3 and sample 4, respectively, corresponding to 10.9% and 7.9% hemolysis, respectively, they also show different degrees of hemolytic activity.

### 3.7 Virtual screening analyses

One potential method for finding new herbal medicines to treat cancer is computer-aided drug design (CADD). The 3D structures of the TCDK4, EGFR, RAS, BRAF, PTEN, and PI3K proteins were created using homology-based modeling.

To set up an already produced protein model with secondary and tertiary structures, begin by retrieving the protein’s 3D coordinates or structural file in PDB format. We utilize molecular visualization software like PyMOL to visualize the protein structure and then prepare it by addressing missing atoms, assigning charges, and performing energy minimization using tools AutoDockT. Starting from the natural components of the plants, the virtual evaluation found the top 15 results from the ZINC Database. Crucial chemical features required for interactions with target proteins were discovered through pharmacophore modeling. We also conduct structural analyses, including secondary structure classification with DSSP assessments. We perform molecular dynamics simulations using GROMACS to explore pharmacophore modeling fits using AutoDock for drug discovery or protein-ligand interaction studies. The development of new small molecule inhibitors for TCDK4, EGFR, RAS, BRAF, PTEN, and PI3K can be guided by these pharmacophore models, opening up exciting possibilities for cancer treatment as mentioned in Table 8.

**TABLE 8:** Virtual Screening and Pharmacophore Modeling for Targeting Cancer-Related Proteins and their Receptors.

Target proteins	Receptors	3D structure	Pharmacophore fit
TCDK4	2I3B	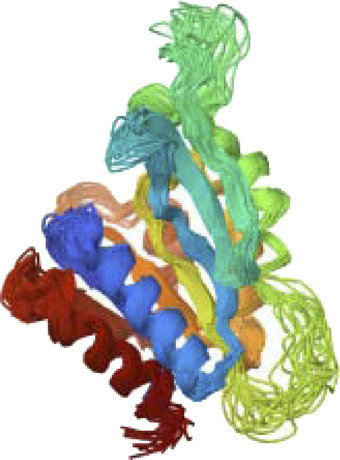	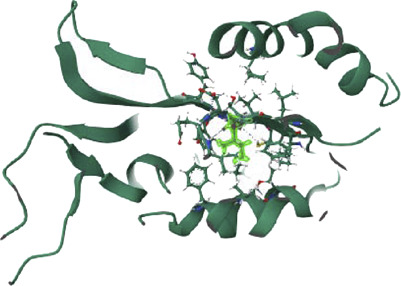
EGFR	3G5X	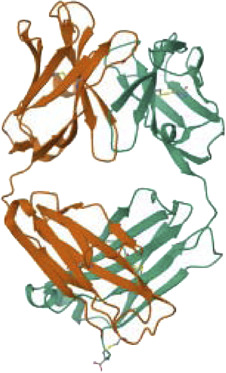	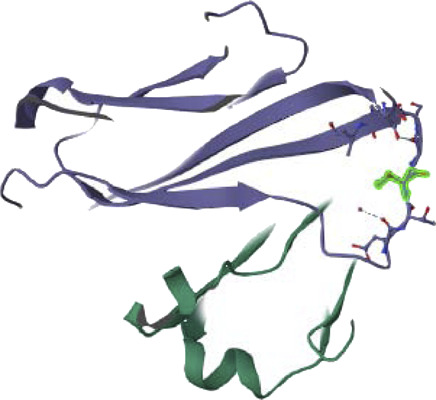
RAS	6T51	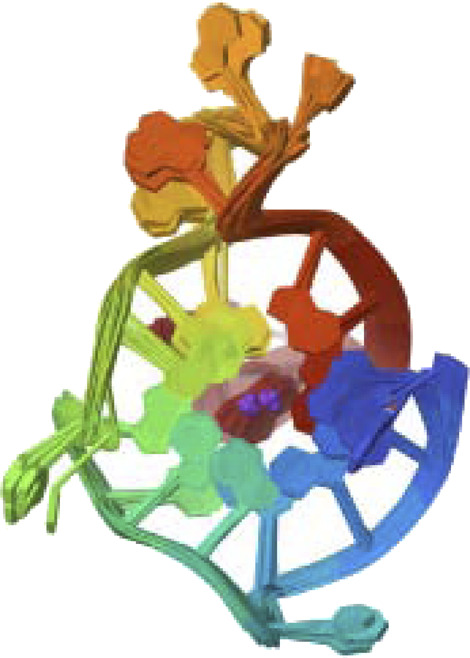	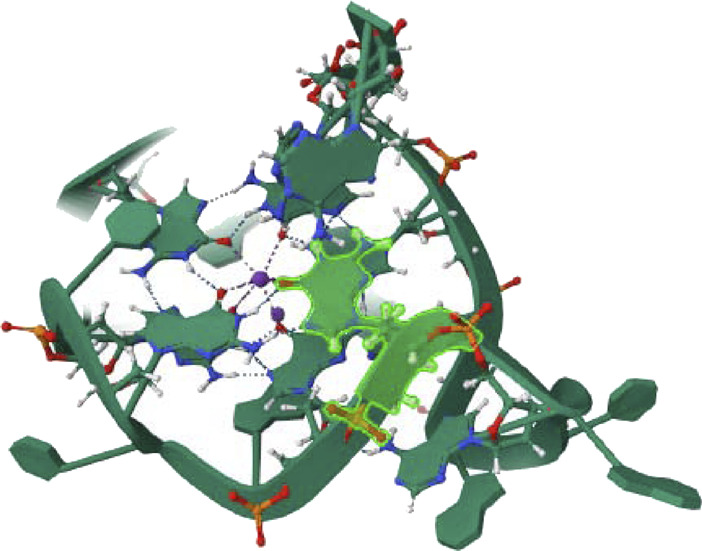
BRAF	4R5Y	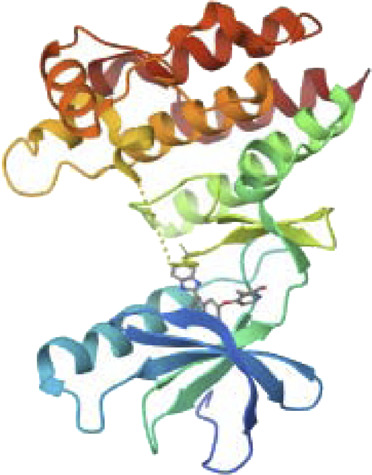	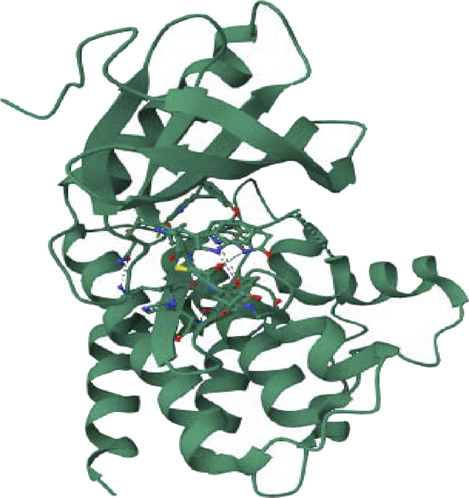
PTEN	2N7X	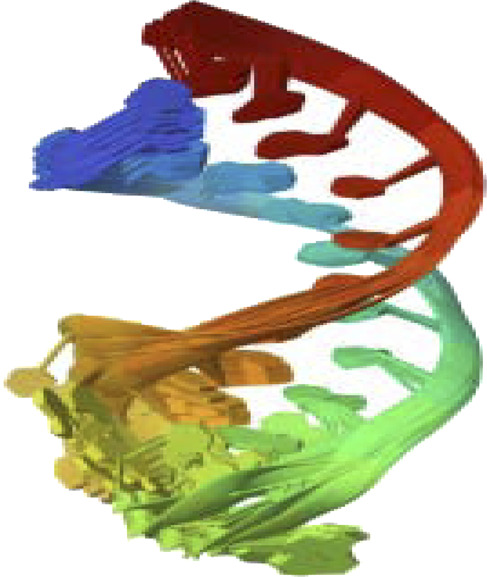	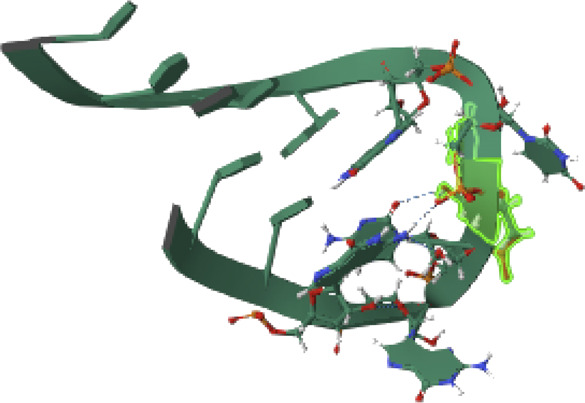
PI3K	6CU6	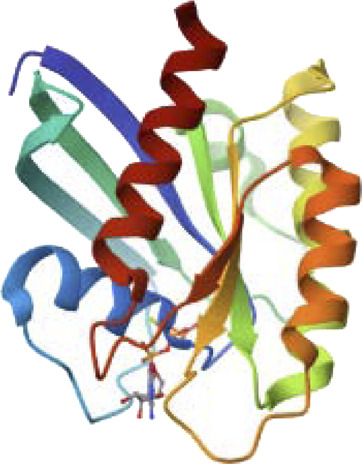	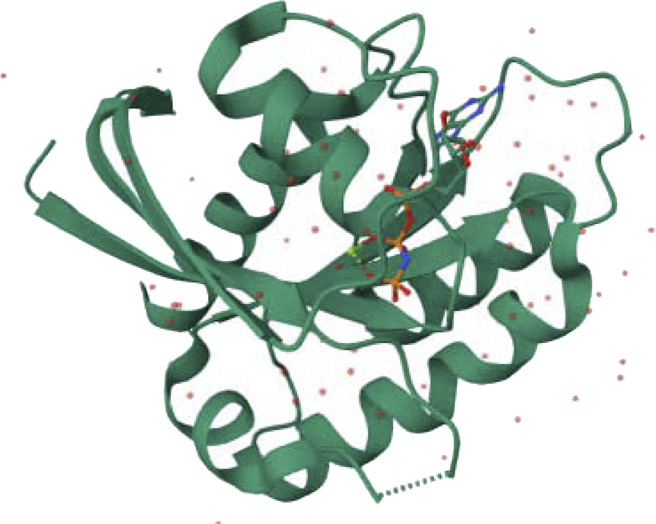

The 3D configuration of chemical properties required for a molecule to interact with a particular biological target is called a pharmacophore. The top 15 hits are small compounds predicted to bind based on their structure, electrostatic properties, and other properties in a virtual assessment of EGFR, BRAF, and PI3K proteins. According to previous research, the surfaces of these proteins include certain nooks and crannies that are necessary for interactions. The pharmacophore alignment scores of the selected compounds were promising, with compound 1 receiving the highest score (53.92) as shown in Table 9. Overall, the pharmacophore of these lead hits probably allows them to interact with some groovesnooks, and crannies on the surface of the protein.

**TABLE 9:** Pharmacophore of top 15 lead hits from virtual screening analyses.

Sr#	Compound	Molecular formula	Rf value	P-value	Molecular weight (g/mol)	Virtual screening Hits	Highest score (%)
1	Thymoquinone	C10H12O2	0.93	0.0001	164.21	53.92	55
2	Thymohydroquinone	C10H14O2	0.90	0.0005	166.23	51.05	51
3	Dithymoquinone	C20H24O2	0.90	0.001	296.42	51.09	51
4	Nigellidine	C12H18N2O	0.90	0.002	206.29	51.07	51
5	Alpha-hederin	C42H72O14	0.92	0.0003	784.99	51.78	52
6	Carvacrol	C10H14O	0.90	0.0002	150.22	50.75	51
7	Nigellimine	C20H24N2O2	0.90	0.0015	324.43	51.06	51
8	Nigellidine-4-O-sulfite	C12H18N2O4S	0.90	0.0023	274.38	51.07	51
9	Melanthin	C18H16O7	0.92	0.0009	328.32	51.80	52
10	Beta-sitosterol	C29H50O	0.90	0.0004	414.71	50.75	51
11	Isoquinoline alkaloids	C9H7N	0.85	0.0012	129.16	50.41	50
12	Linoleic acid	C18H32O2	0.85	0.0008	280.45	50.23	50
13	Oleic acid	C18H34O2	0.81	0.0006	282.46	47.34	47
14	Palmitic acid	C16H32O2	0.75	0.0009	256.43	45.34	45
15	Myristic acid	C14H28O2	0.85	0.0003	228.36	50.02	50

A pharmacophore is a diagram that represents the configuration of the functional groups that give a molecule its biological action. From virtual screening, the research produced a pharmacophore model for the top 50 lead hits. High pharmacophore scores (from 50.75 to 53.92), indicating a strong match to the pattern and potential for strong binding to the target protein, were used to select the top 15 compounds. However, high pharmacophore scores do not guarantee the effectiveness of a drug. More research, such as molecular docking and dynamic simulations, is needed to assess the compounds’ ability to bind and remain stable as drugs.

### 3.8 Molecular docking

The ligand Neuropilins and the active receptor with PDB ID 2I3B were docked to compute binding energy dynamics and detect interactions. Swiss PDB Viewer was used to build the protein structure, and AutoDock Vina software was used for docking. For docking tests, protein structures from the Swiss ADME target prediction site were used. Using the MGLTools program, the protein structure was protonated and given Kollman charges to reflect electrostatic characteristics as part of the ligand production process.

Rather et al. (2020) technique was used in several docking simulations to find the protein receptors with the lowest ligand binding energy values. Several factors were examined, including Ligand rmsd, Receptor LGscore, Receptor MaxSub, Ligand LGscore, and Ligand MaxSub. The protein receptors 2I3B, 3G5X, 6T51, 4R5Y, 2N7X, and 6CU6 received docking scores showing their affinities with the ligand. Confidence ratings for receptor interactions ranged from 0.8562 to 0.9366. Higher confidence values mean that the docking findings are more reliable, but lower docking scores indicate stronger binding. The results provide information about the interactions and binding affinities between the ligand and the protein receptors.

In Table 10, the quality parametric analysis of the input model using ProQ v1 is examined, with a focus on the interactions of the receptor and ligand with the protein. To evaluate the merits of protein models, the LGscore and MaxSub ranges are established. Correct outcomes vary from 1.5 to LGscore and 0.1 to MaxSub, while excellent values range from 5.0 to LGscore and 0.8 to MaxSub. Good results range from 3.0 to 5.0 for LGscore and 0.5 to 0.8 for MaxSub.

**TABLE 10:** Molecular Docking with Centromere-related protein targets and their parametric scores.

Complexes	Receptors	Docking score	Confidence score	Ligand RMSD (Å)	Receptor LGscore	Receptor MaxSub	Ligand LGscore	Ligand MaxSub	Binding affinity
CDK4	2I3B	−253.19	0.8873	55.29	6.197	0.537	5.244	0.334	High
EGFR	3G5X	−284.64	0.9366	92.52	5.474	0.452	5.244	0.334	Very High
RAS	6T51	−243.71	0.8669	137.79	3.919	0.372	5.244	0.334	Medium
BRAF	4R5Y	−254.96	0.8908	94.29	5.640	0.514	5.244	0.334	High
PTEN	2N7X	−250.34	0.8815	77.14	2.014	0.261	5.244	0.334	Medium
PI3K	6CU6	−239.19	0.8562	64.80	4.271	0.489	5.244	0.334	High

A monoclonal antibody called Neuropilins that targets the growth factor VEGF is used to treat cancer (Bergsland, 2004). A protein called CDK4 is involved in the control of cell growth and division. According to Nwaefulu et al. (2022) EGFR, a cell surface receptor that controls cell growth and division, is overexpressed in a variety of malignancies. RAS proteins are essential for cell signaling and development because they bind to Wnt proteins (Wesslowski et al., 2020). BRAF is a receptor protein that plays a role in cell survival and proliferation, and aberrant activation has been related to the onset and spread of cancer.

**FIGURE 5:**
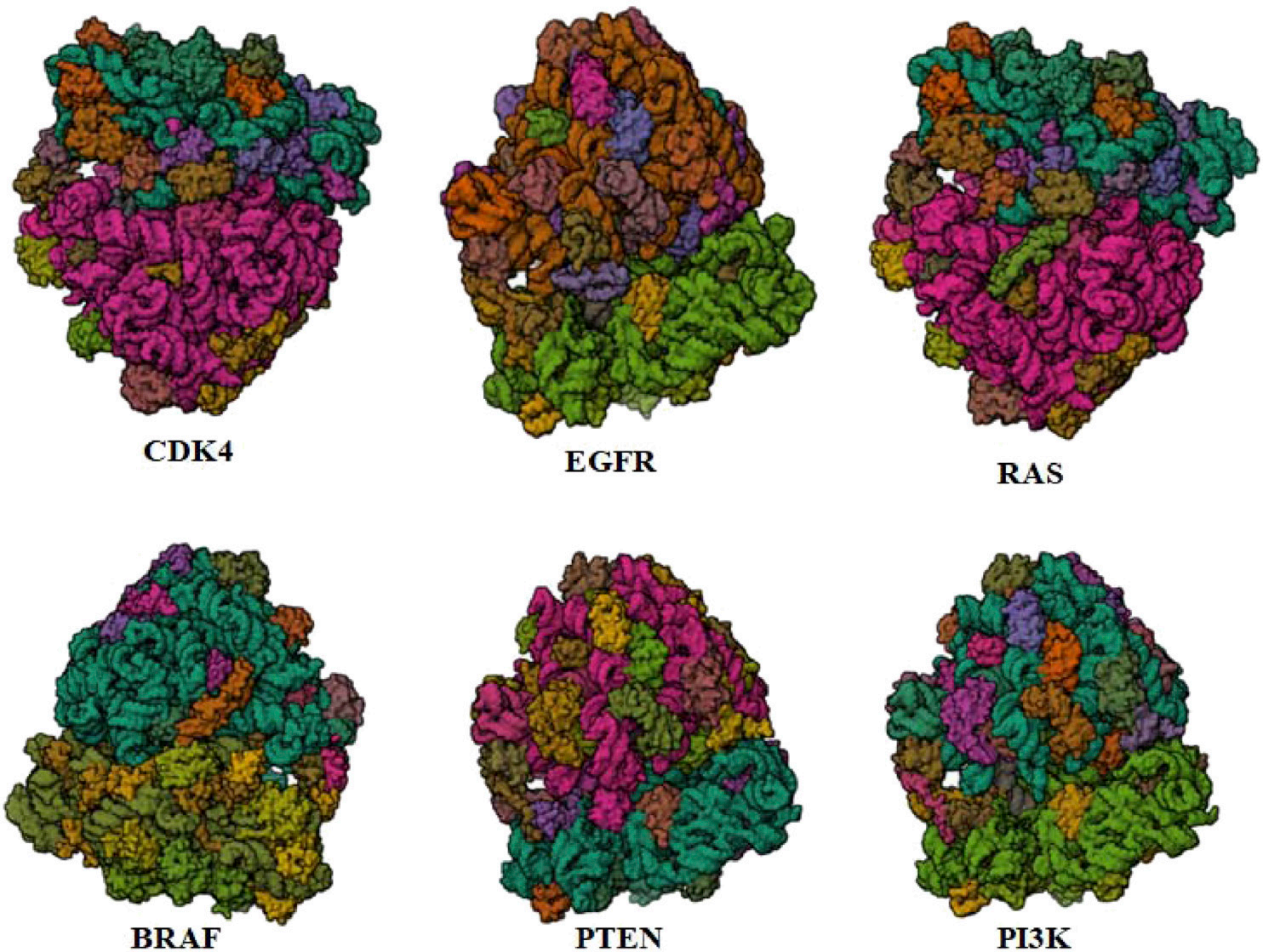
Neuropilins embedded in the active sites of CDK4, EGFR, RAS, BRAF, PTEN, and PI3K proteins.

PTEN is a receptor tyrosine kinase that is connected to certain cancers, such as gastrointestinal stromal tumors (GISTs), and is involved in cell signaling (Tsai et al., 2022). Cancer patients often have active PI3K, a synthetic transcription factor that mimics the function of an oncogene (Dhanasekaran et al., 2022). While they do not directly interact with CDK4, EGFR, RAS proteins, BRAF, PTEN, or PI3K in their active areas, neuropilins predominantly target VEGF. Each protein performs and interacts in a unique way (Figure 5).

The docking score of −253.19 for the CDK4 complex shows that Neuropilins and CDK4 have a significant affinity for one another, which is supported by findings from previous studies (Marriam et al., 2020). With a confidence level of 0.8873, docking findings seem to be trustworthy. The RMSD of the ligand, 55.29, indicates a considerable conformational variation. The quality of the CDK4 receptor model is shown by the LGscore of 6.197 and the MaxSub of 0.537, while the quality of the ligand model is indicated by the LGscore of 5.244 and the MaxSub of 0.334 for Neuropilins. With a confidence score of 0.9366 and a docking score of −284.64 for the EGFR complex, it is clear that Neuropilins and EGFR have a significant attraction for one another. With a ligand RMSD of 92.52, there is a substantial conformational deviation.

**FIGURE 6:**
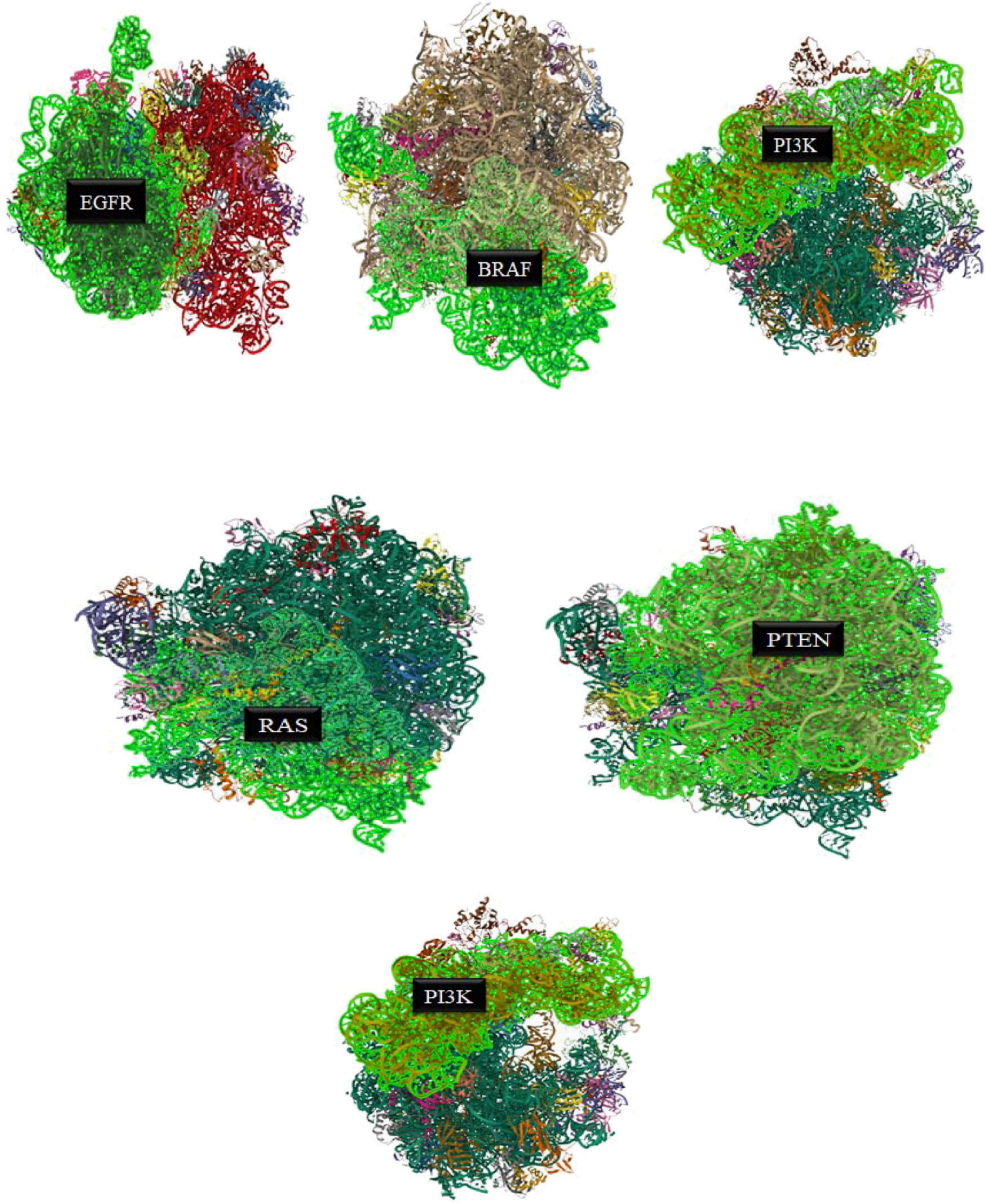
EGFR, BRAF, PI3K, RAS, PTEN, and PI3K complexes with Neuropilins.

The quality of the EGFR receptor model is shown by the LGscore of 5.474 and MaxSub of 0.452, while that of the ligand model by Neuropilins is shown by the LGscore of 5.244 and MaxSub of 0.334. As can be shown in Figure 6, the results support the significant binding affinities between Neuropilins and both CDK4 and EGFR complexes. The quality parameters for the receptor and ligand models are adequate and support the docking simulations’ claims of reliable interactions.

With a confidence score of 0.8669 and a docking score of −243.71 for the RAS complex, it is clear that Neuropilins and RAS have a significant affinity for one another. RMSD for the ligand is 137.79, which suggests a significant conformational variance. The quality of the RAS receptor model is shown by the LGscore of 3.919 and the MaxSub of 0.372, while the quality of the ligand model for Neuropilins is reflected by the LGscore of 5.244 and the MaxSub of 0.334. The docking score of −254.96 for the PTEN complex reveals that Neuropilins and PTEN have a high affinity for one another. While the ligand RMSD of 94.29 shows large conformational variability, the confidence score of 0.8908 indicates very credible data. The quality of the PTEN receptor model is shown by its LGscore of 5.640 and MaxSub of 0.514, while that of the Neuropilins ligand model is reflected by its LGscore of 5.244 and MaxSub of 0.334. As can be shown in Figure 6, the findings support the significant binding affinities between Neuropilins and both RAS and PTEN complexes. Despite ligand RMSD values indicating structural variations, the docking simulations show reliable connections. The credibility of the docking findings is supported by the fact that the quality parameters for the receptor and ligand models are within acceptable limits.

The PTEN signaling pathway complex (2N7X) and Neuropilins have a reasonably significant binding affinity, according to the docking score of −250.34. A solid docking result is suggested by the confidence score of 0.8815. A substantial variation in the structure of the ligand is indicated by the ligand RMSD of 77.14. The PTEN receptor model’s receptor LG-score of 2.014 and MaxSub of 0.261 indicate good quality. For Neuropilins’ Complex, a Ligand LG-score of 5.244 and MaxSub of 0.334 indicate high quality. Overall, the findings point to a robust binding relationship, with trustworthy docking and favorable receptor-ligand model characteristics (Figure 6).

Neuropilin interactions with CDK4, EGFR, RAS, BRAF, PTEN signaling pathway, PI3K, and 6DRY are shown in Figures 3 to 6. PyMOL was used to visualize 3D data, while BIOVIA Discovery Studio was utilized to investigate 2D interactions. The combination of these technologies offers useful insights into the specifics of Neuropilins’ structural makeup and prospective therapeutic uses.

Three natural compounds, Compound-1, Compound-2, and Compound-3, showed the greatest binding affinities and significant molecular interactions in the docking study of the top 15 lead hits against the P53 protein. These substances had exceptional binding energies that ranged from −11.2 to −12.9 kcal/mol, and they created vital connections with important residues in the targeted P53 protein’s active areas. The remaining chemicals were found to have weaker interactions with the protein and lower binding energies. The results mentioned in Table 11 highlight the possibility of these top three natural chemicals as viable possibilities for the creation of brand-new cancer treatment medications. The discovery of powerful binding sites and widespread molecular interactions in the docked complexes provides crucial knowledge for the development of efficient P53 protein inhibitors.

**TABLE 11:** Binding affinities of top 3 natural compounds against P53 protein.

Compounds	Binding affinities (kcal/mol)
Compound-1	−12.9
Compound-2	−11.6
Compound-3	−11.2

These findings demonstrate Compound-1’s greater binding affinity, which earned it the top docking score of −12.9 kcal/mol. Compound-2 came in second with a binding energy of −11.6 kcal/mol, while Compound-3 was third with a binding energy of −11.2 kcal/mol. Notably, all three substances revealed positive interactions with essential residues found in the P53 protein’s active regions, offering a compelling case for their potential as powerful therapeutic candidates.

With molecular weights below 500 g/mol, 10 hydrogen acceptors, 5 hydrogen donors, and 5 LogP values, suggesting acceptable bioavailability and membrane permeability, the top three hits were evaluated using Lipinski’s rule of five. This revealed that they fulfill important drug-likeness requirements. These substances also show enough amounts of hydrogen bond acceptors, donors, and rotatable bonds, indicating potential pharmacological action. Table 12 summary of the physicochemical features of the top three hits shows that they match those of medications and have the potential to be strong contenders for anticancer drugs. They are also quickly absorbed by the gut and are non-carcinogenic, which further supports their suitability for use in the creation of anticancer drugs, according to the ADMET study.

**TABLE 12:** The analyses of physicochemical Properties of the top 3 selected compounds.

Properties	Compound-1	Compound-2	Compound-3
Molecular Weight	425.39 g/mol	413.40 g/mol	495.72 g/mol
LogP (o/w)	2.06	3.90	9.02
H-bond Acceptors	6	6	2
H-bond Donors	3	3	2
Rotatable Bonds	5	5	1
PSA	74.92 Å^2^	92.05 Å^2^	30.23 Å^2^
Atoms	54	50	91
Rings	5	4	6
Bioavailability	✔ Yes	✔ Yes	✔ Yes
Intestinal Absorption	✔ Yes	✔ Yes	✔ Yes
Carcinogenicity	✖ No	✖ No	✖ No

### 3.9 Structure assessment

**FIGURE 7:**
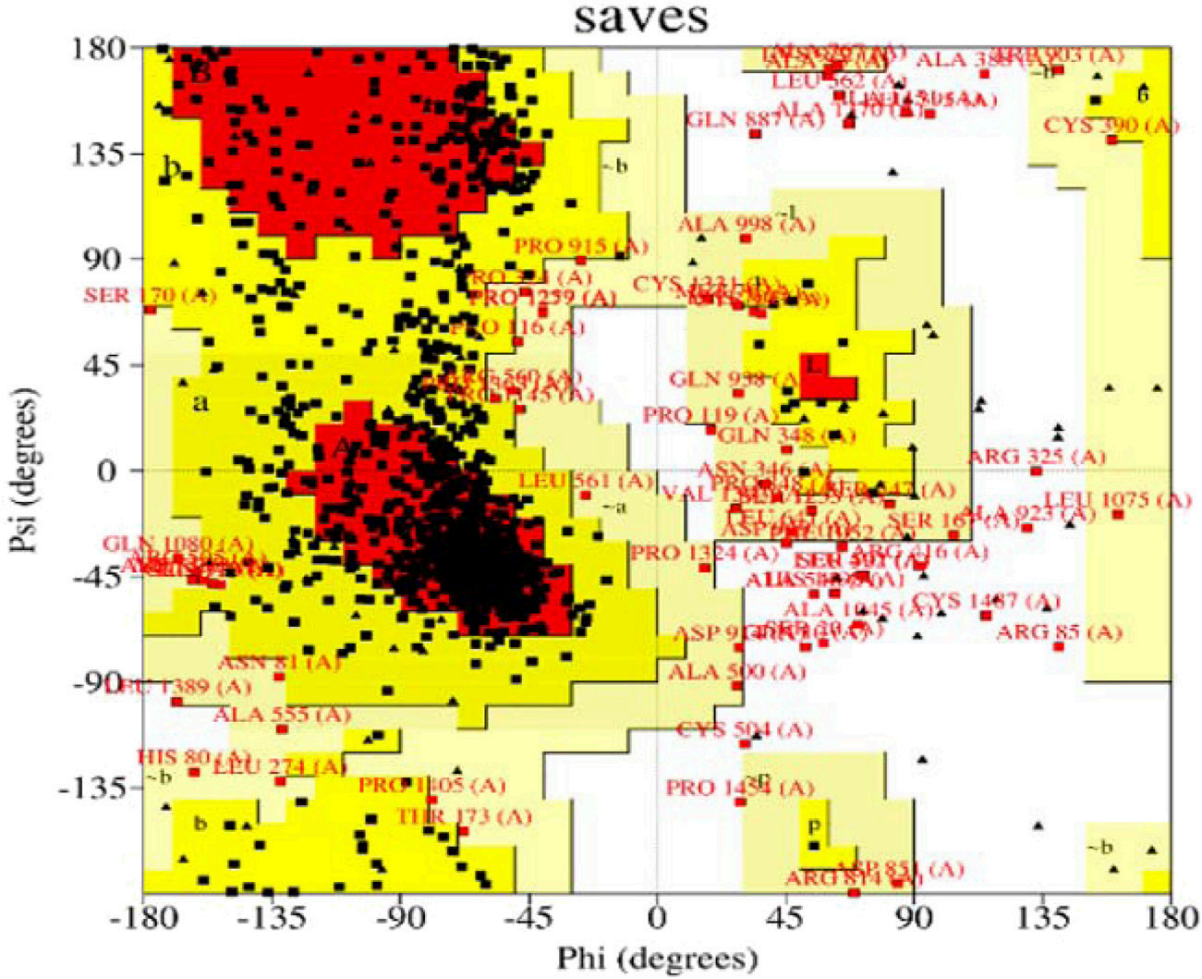
Structure assessment of PI3K by using Ramachandran plot.

We used the Ramachandran approach to illustrate the allowed regions of amino acid backbone dihedral phi *versus* psi angles in PI3K protein chain-A which strongly bind with Neuropilins as explored in the docking process. It provides information about the stereochemical properties of a protein and is usually generated using software such as PROCHECK. We used PROCHECK software to generate a Ramachandran plot for PI3K. Figure 7 shows the Ramachandran plot of PI3K, showing the distribution of residues in different regions. The graph shows that 73.1% of residues are in highly favored regions, 21.6% in extra allowed regions, 3.0% in generously allowed regions, and 1.8% in forbidden regions. Specifically, the graph reveals that of the total 1,232 non-glycine and non-proline residues, 907 (73.6%) are in the most favored regions, 266 (21.6%) in additional allowed regions, 37 (3.0%) in generously permitted regions and 22 (1.8%) in disallowed regions. The graph also includes 122 proline residues and 143 glycine residues, which are represented separately.

The Ramachandran diagram serves as a visual representation in protein structure analysis to evaluate the conformations of protein residues in three dimensions. It provides valuable information about the orientation of amino acid side chains in space through the phi and psi angles. In the case of PI3K, the Ramachandran diagram was used to evaluate the protein’s stereochemistry and determine whether it adopts an appropriate and stable conformation. In addition, the graph helps identify residues that may be in unfavorable positions, which may affect protein folding or stability. This information is critical for optimizing protein expression, stability, and function.

### 3.10 Molecular dynamic simulations

Due to their high binding affinities, the top-chosen PI3K complexes underwent MD simulations. The stability and consistency of the complexes were evaluated using the gmx-rmsd module of GROMACS, a popular program for molecular dynamics simulations. The PRODRG topological file was used to create the ligands, guaranteeing correct parameterization for use in the simulations. Using RMSD values derived from the simulations, this work sought to analyze the dynamics and structural changes of the complexes over time (Van Der Spoel et al., 2005).

### 3.11 Binding stability of protein-ligand interactions

The research used MD simulations to examine the binding stability of the top Neuropilins complex with its corresponding ligand (6CU6). At room temperature, the simulations were run for 200 ns. The study took into account the average center of mass distance between the protein and ligand as well as RMSF, RMSD, and hydrogen bonding. The findings provide insightful information on the dynamics, stability, and binding properties of each Neuropilins complex with its ligand (6CU6). The reliability and precision of the MD simulations are confirmed by comparing the computed parameters with experimental data, proving the stability and binding interactions of the complexes.

### 3.12 RMSF analysis

**FIGURE 8:**
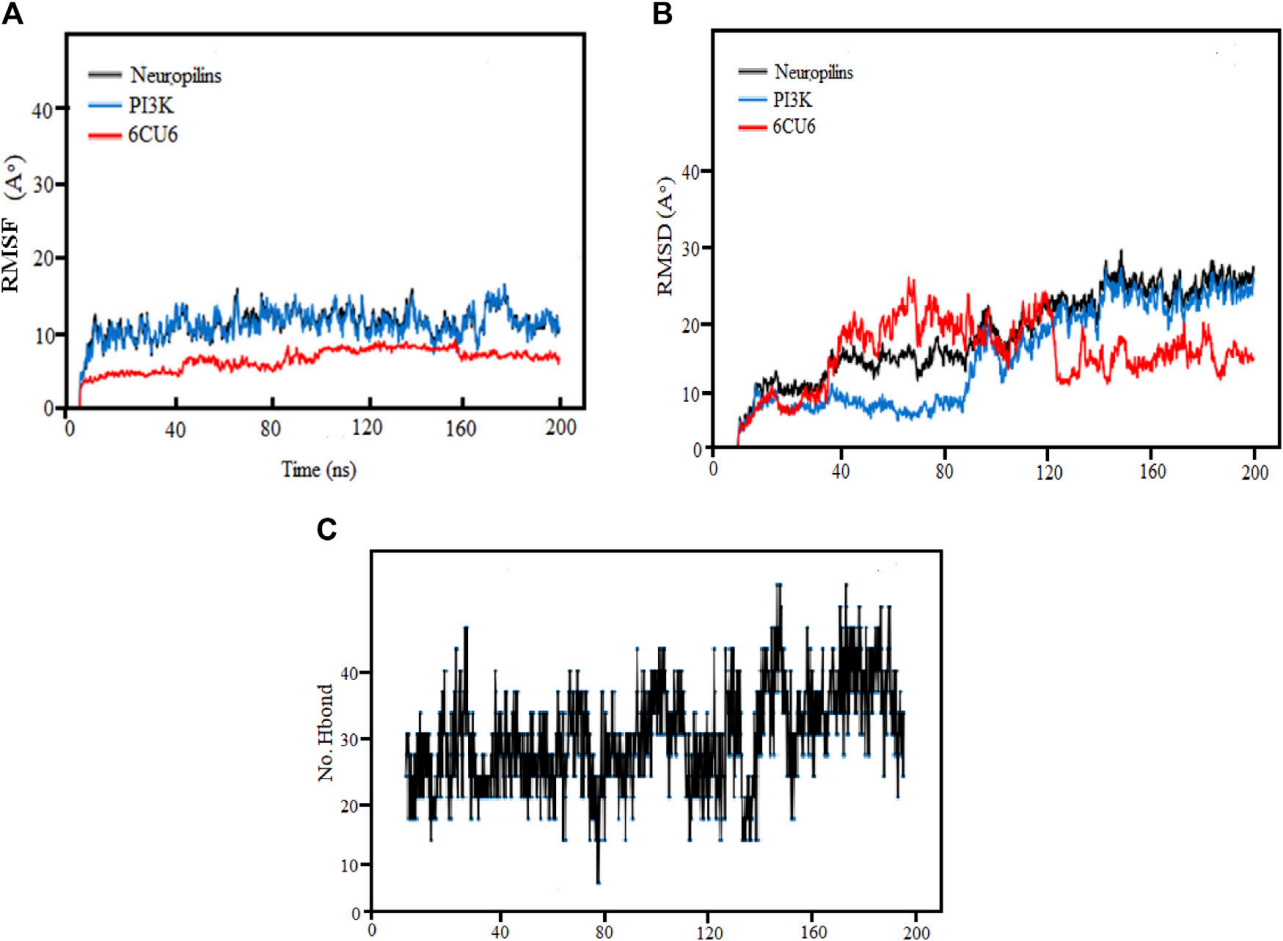
**(A)** Demonstration of RMSF values, **(B)** RMSD Analysis and **(C)** Hydrogen Bonds for the complexes (PI3K), (Protein-ligand) during 200 ns MD simulation.

By assessing the Root Mean Square Fluctuation (RMSF) originating sites, we could ascertain whether any significant changes had occurred in the target molecule. As depicted in Figure 8A, we observe diverse patterns in the RMSFs of the observed complex, protein, and ligand for each molecule. Notably, the RMSDs of the PI3K complexes for both the complex and protein exhibited remarkable stability, with only minor fluctuations. The average RMSF values for the complex, protein, and ligand were found to be 3, 10, and 13, respectively, indicating that most variations occurred between 40 ns and 200 ns. After the initial 20 ns, Complex PI3K displayed a consistent fluctuating pattern of RMSF with minimal variation, maintaining a nearly constant behavior throughout the experiment.

Consistent with the figure’s depiction of relatively stable complexes, those with stronger bonding and higher stability exhibited lower RMSF values and reduced volatility, as evident in the Neuropilins and PI3K complexes. The RMSF analysis of backbone atoms within the PI3K complexes significantly enhanced our understanding of the flexibility of specific residues within these complexes.

### 3.13 RMSD analysis

Root mean square deviation (RMSD) analysis serves as a powerful tool to understand the dynamic nature and fluctuations within protein complexes. In this research, we performed 200 ns molecular dynamics (MD) simulations on different PI3K complexes to explore their conformational dynamics.

Throughout the simulations, we identified specific regions with elevated RMSD values, indicating greater flexibility. Meanwhile, most residues maintained stable conformations, exhibiting RMSD values below the threshold of 30. It was notable that complexes exhibiting RMSD values greater than 80 showed more pronounced variability and greater sensitivity to conformational changes. In particular, some residues showed increased flexibility during the initial 120 ns, followed by reduced fluctuations from 160 ns to 200 ns, ultimately leading to a more stabilized structure.

The RMSD analysis further revealed temporal fluctuations in protein conformation during the simulations from 1 to 2.8^A^. In addition, the structural changes within the complexes concerning their initial configurations were elucidated. Although both the protein and the ligands underwent significant changes and decreased stability during the simulation, the neuropilin compound maintained remarkable stability between 120 and 200 ns, as visually shown in Figure 8B along with PI3K.

The simulated proteins showed typical thermodynamic behaviors. Initially, RMSDs increased due to system easing based on stable trajectories around their mean values for more than half of the simulation runs. It is worth noting that the RMSD of ligand-bound proteins remained consistently lower and less variable compared to unliganded proteins (ranging from 1 to 2.8 Å). This dynamic behavior implied greater compactness and stability when proteins were bound to ligands. Crucially, all holo hiMGAM proteins eventually converged around an average RMSD of 1.05 ± 0.30 Å in more than half of the 120 ns simulation runs, demonstrating the validity of the simulation without requiring a time-out longer simulation.

**FIGURE 9:**
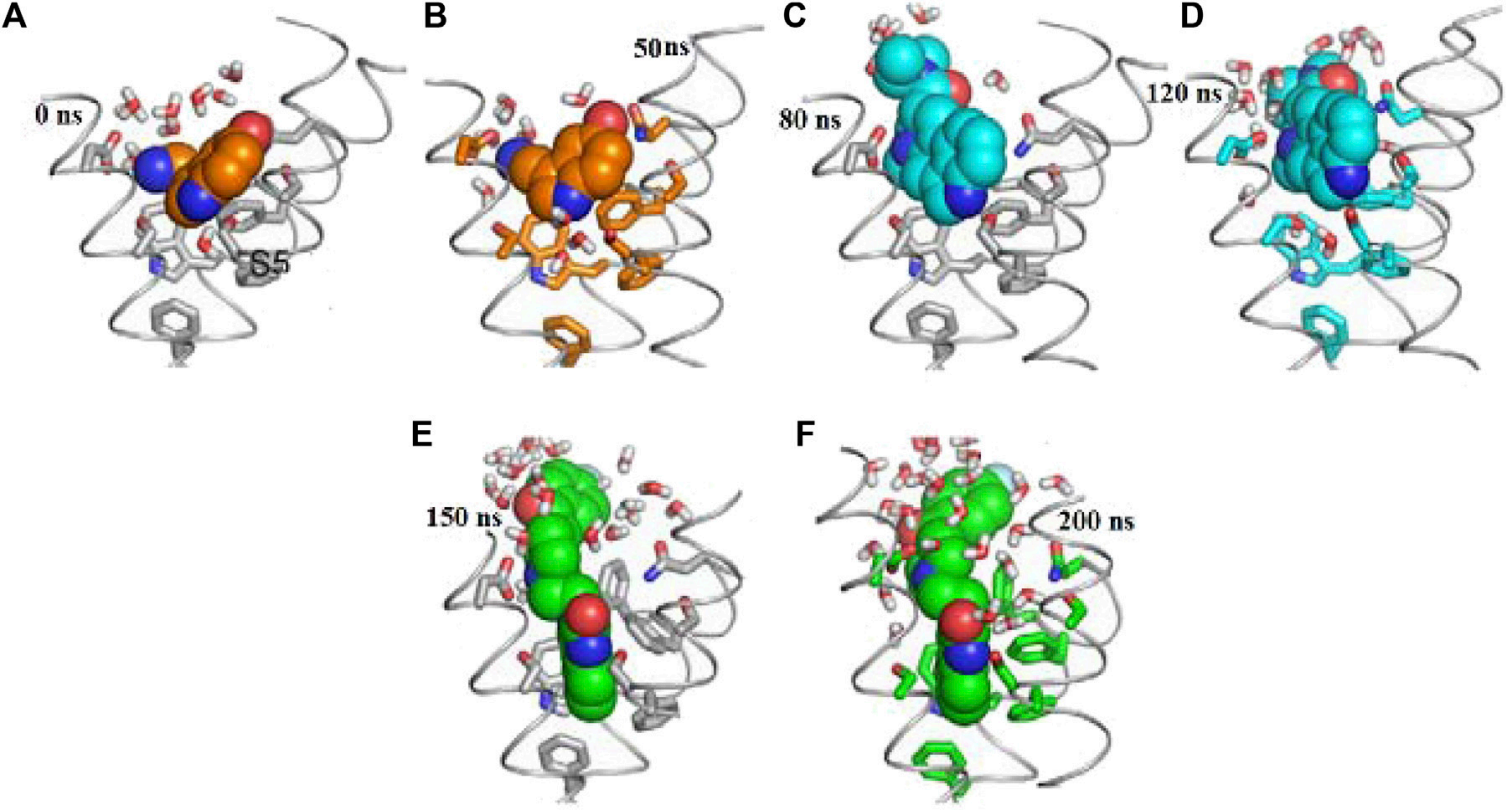
Docking possesses **(A–F)** overlaid ligand snapshots at initial and final timeframes: **(A)** Complexes-0, **(B)** C-50, **(C)** C -80, **(D)** C -120, **(E)** C -150, **(F)** C -200. Ligands (sticks) and bounded proteins (bolls) presented with different color codes concerning 0 ns and 200 ns extracted frames.

We explore RMSD of individual ligands, outstanding stability was observed within the target protein binding sites for the simulated ligands at different time frames (C-0, C-50, C-120, C-150, and C-200), as shown in Figure 9, with variations of different Å values and results where compared and followed by Elhady et al. (2023). These simulations showed minimal fluctuations and nearly constant trajectories compared to other ligands. The C-0 and C-50 ligands showed lower RMSD values, indicating confinement in the ligand binding pocket and minimal conformational changes during the simulations. In contrast, other ligands exhibited higher RMSD values and significant fluctuations (approximately 2.8 ± 0.50 Å) during specific time intervals, indicating orientational changes and reduced stability of the ligand–target complexes. However, these less stable models eventually reached a constant RMSD plateau with an average value after 200 ns of simulation time. Importantly, the RMSD of all simulated ligands never exceeded three times the values observed when bound to target proteins after reaching their respective simulation levels. This confirms the significant presence of ligands in the target pocket, which ensures complex stability and successful protein convergence.

### 3.14 Ligand binding modes analysis

We analyzed the ligand binding modes for the N. sativa-isolated compound and identified crucial hydrogen bonding interactions. These interactions play a significant role in ligand binding affinity and specificity. The details of the protein-ligand complexes for hydrogen bond interactions that developed between the protein and ligand during the 200 ns MD simulation are shown in Figure 8C. These interactions are crucial for the stability of complexes and binding affinity. The average separation between the ligand and the protein throughout the simulation provides details on the ligand’s spatial organization and dynamic behavior in the binding pocket. In the main context of Hydrogen Bonding Analysis, Our results revealed prominent polar hydrogen bonds between the ligand and the protein binding pocket. These interactions were found to be essential for stabilizing the ligand within the pocket. We calculated the distances between hydrogen bond donors and acceptors in the identified hydrogen bonds. The average hydrogen bond distance was found to be approximately 2.5 Å, indicative of strong hydrogen bonding interactions. The angles formed by hydrogen bonds were also examined. These angles provide insights into the geometry of hydrogen bonding interactions. The average hydrogen bond angle was approximately 120°, indicating a favorable geometry for hydrogen bonding. In addition to hydrogen bonds, we assessed the presence of hydrophobic contacts between the ligand and the protein binding pocket. Hydrophobic interactions are known to contribute to ligand binding stability. Our analysis revealed significant hydrophobic contacts, further enhancing the ligand’s binding affinity.

**FIGURE 10:**
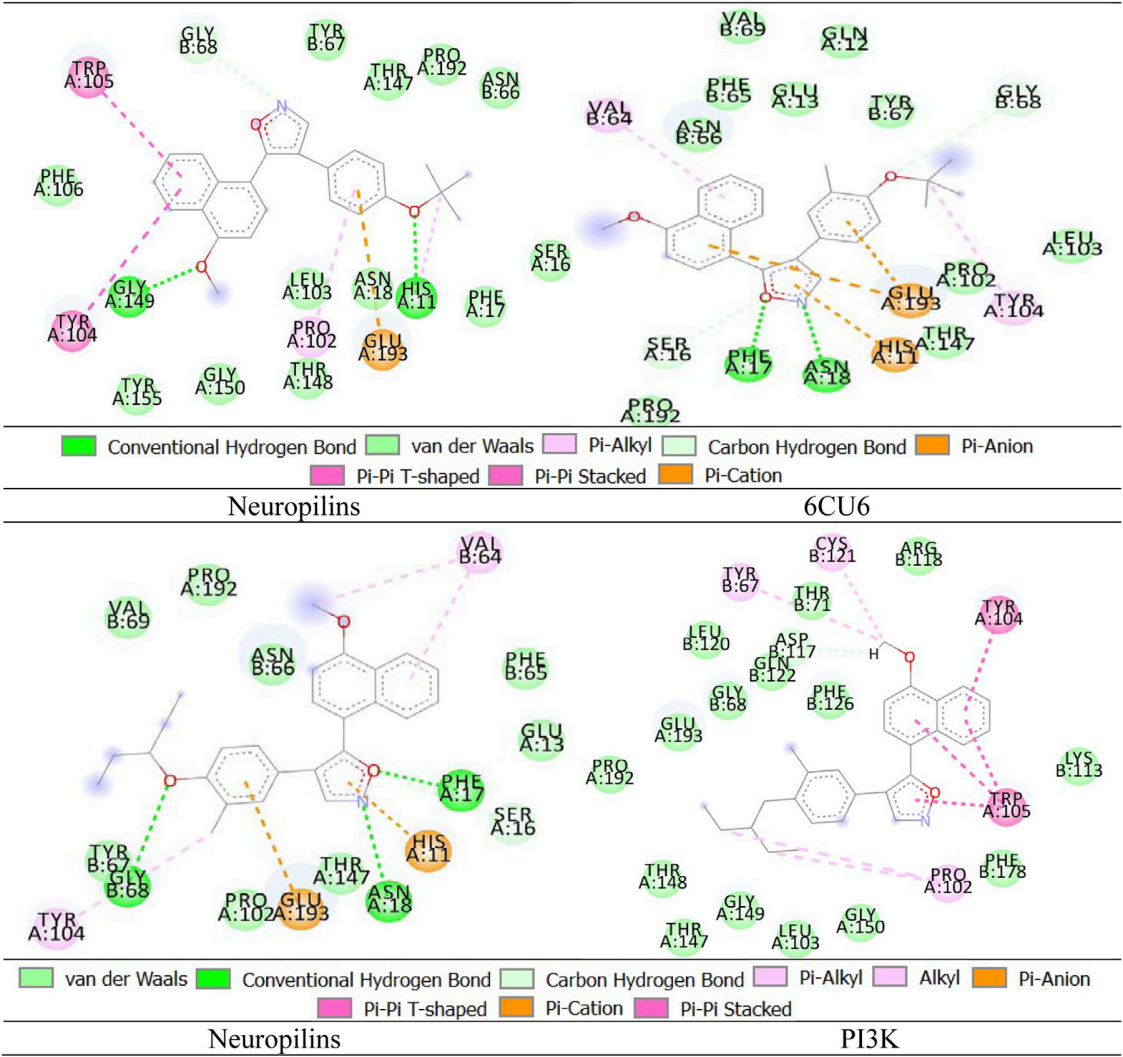
Different interactions of proposed compounds.

We enhance our understanding of protein-ligand interactions and complex stability during MD simulations by analyzing hydrogen bond patterns and average distance values. These specific insights play a crucial role in assessing binding kinetics and affinity, furnishing valuable data for further analysis and interpretation. The results indicate that all the examined compounds interact with amino acids in the same pocket of the PI3K receptor’s active site. Moreover, these compounds exhibit a significant affinity compared to Neuropilins, forming numerous binding interactions with the 6CU6 receptor, as demonstrated in Figure 10.

For a comprehensive depiction of ligand binding modes, we have generated both 2D and 3D representations of the N. sativa-isolated compound within the protein binding pocket. These representations elucidate the spatial orientation of the ligand and its interactions with the adjacent residues. The 2D binding pose highlights crucial interactions, such as hydrogen bonds and hydrophobic contacts, while the 3D binding pose offers a three-dimensional perspective of the ligand’s positioning within the pocket.

### 3.15 MM-GBSA analysis

Using the molecular dynamics (MD) simulation trajectory, we determined the binding free energy and associated energy components for the Neuropilins-PI3K complex through the MM-GBSA method as mentioned in Table 13. Our analysis revealed that the primary contributors to ΔGbind, which stabilizes the simulated complex, are ΔGbindCoulomb, ΔGbindvdW, and ΔGbindLipo. In contrast, ΔGbindCovalent and ΔGbindSolvGB contribute to the instability of the complex (Mukerjee, 2022). These findings underscore the high binding free energy observed for the Neuropilins-PI3K complex, indicating its efficient binding capacity and ability to form a stable complex with Neuropilins. This analysis provides valuable insights into the molecular energetics and stability dynamics of this interaction.

**TABLE 13:** MM-GBSA analysis of Neuropilins-PI3K binding energies.

Compound code (ns)	MM-GBSA (kcal/mol)					
	ΔG_bind_	ΔG_bind_Lipo	ΔG_bind_vdW	ΔG_bind_Coulomb	ΔG_bind_SolvGB	ΔG_bind_Covalent
0	−10.41	−6.82	−17.93	−12.05	14.40	0.001
20	−10.15	−5.85	−17.17	−12.83	15.31	−2.604
40	−11.96	−6.58	−16.33	−1.67	4.33	2.290
60	−11.31	−5.94	−13.08	−3.93	6.50	0.155
80	−11.33	−5.48	−18.00	−4.55	10.63	1.063
100	−11.53	−9.73	−12.18	−14.69	17.03	0.048
120	−12.92	−6.70	−15.21	−7.27	14.75	8.512
140	−12.89	−6.68	−13.83	−9.62	15.42	8.819
160	−13.32	−10.77	−13.90	−4.02	9.081	7.240
180	−12.62	−8.52	−12.21	−0.92	10.17	2.877
200	−12.31	−3.33	−12.02	−3.96	9.45	−0.451

## 4 Discussion

Breast cancer is a complex disease with multiple oncogenes driving tumor growth. Researchers are exploring a new approach to target these oncogenes using neuropilins (NRPs), which are receptors involved in cancer signaling (Abdullah et al., 2021). To understand this better, we use computer-based in siliconin-silicon studies to predict how NRPs might interact with the oncogenes in breast cancer cells as per the method of (Kumar et al., 2021). Researchers (Rafique et al., 2023; Zafar et al., 2023) analyze gene expression data and simulate molecular interactions to see how NRPs could affect cancer pathways. To validate the findings, we conducted *in vitro* studies in the lab. As in earlier researchers use breast cancer cells in controlled experiments to see how manipulating NRPs affects cancer cell behavior and look at changes in cell growth, movement, and other important functions (Azari et al., 2023).

By investigating the bioactive compounds of *Nigella sativa* and their potential to target neuropilins, this research offers the prospect of novel, more effective, and less toxic therapies for breast cancer patients. Utilizing computational methods for initial screening (*in silico* analysis) accelerates drug discovery, while subsequent *in vitro* validation ensures the practicality of these compounds in real biological settings. We explore the gene and protein expression to understand how NRPs influence the oncogenic signaling in breast cancer cells as per the method of earlier researchers (Yousefi et al., 2021; Campos et al., 2022). The potential of NRPs as therapeutic targets is promising. Insilico studies suggest that targeting NRPs could be an effective way to tackle multiple oncogenes simultaneously and the results were matched with (Abdullah et al., 2021). By combining NRP-targeted therapy with existing treatments, it may improve the overall effectiveness of breast cancer treatment and reduce the risk of drug resistance (Fu et al., 2019).

In this study, we applied the Thin-Layer Chromatography (TLC) technique for the phytochemical screening (Li et al., 2021) and observed their separation based on their distinct Rf (retention factor) values and observed under ultraviolet (UV) light (Engel and Schiller, 2021). The total antioxidant capacity (TAC) assay was performed to assess the ability of NRPs to scavenge free radicals and mitigate oxidative stress (Yang et al., 2009). The reducing power determination was employed to understand NRPs’ potential in electron donation and antioxidant activity. To evaluate the potential toxicity, the hemolytic activity of NRPs was assessed. Using Bioinformatic analyses we predict the protein structure to gain insights into the three-dimensional structure of NRPs as per the method of earlier researchers (Ahmad et al., 2022; Ali et al., 2023a; Ali et al., 2023b). Subsequently, molecular docking was performed to understand the interactions between NRPs and Multi-Coded Oncogenes, elucidating potential binding modes and affinities (Marriam et al., 2020). Furthermore, We performed molecular dynamics simulations to predict valuable information about the dynamic behavior of the NRP-oncogene complexes over time as per the method of earlier researchers (Rather et al., 2020). Lastly, trajectory analysis was performed to analyze the complex’s stability and infer the key residues involved in the binding interactions.

We observed the presence of phytochemical tannins, saponins, steroids, and cardiac glycosides, while phlobatannins and terpenoids were absent as in the same context of earlier researchers (Ali et al., 2023b). Quantitative estimation of the crude chemical constituents in *N. sativa* leaves showed an alkaloid content of 9.4% ± 0.04% and saponins at 1.9% ± 0.05% and results were matched with (Ali et al., 2023c). The percentage yield of plant extracts varied with the solvent used, with methanol having the highest yield (4). Methanol extract also displayed the highest Total Flavonoid Content (127.51 ± 0.76 mg/100 g), while n-hexane had the lowest (4.47 ± 0.05 mg/100 g). Methanol extract also exhibited the highest. Total Phenolic Content (134.39 ± 0.589 mg GAE/100 g), while n-hexane had the lowest (23.25 ± 0.102 mg GAE/100 g). The Total Antioxidant Capacity (TAC) assay showed a concentration-dependent antioxidant effect of *N. sativa* seed extract. The reducing power of *N. sativa* leaf extracts was higher at higher concentrations. Hemolytic activity testing revealed varying degrees of hemolysis for different *N. sativa* leaf extracts. Positive control (0.1% Triton X-100) induced 100% hemolysis, while different extracts showed 3.7%–16.5% hemolysis.

In the framework of the study mainly we evaluate *N. sativa* compounds for their potential to attack neuropilins, it was also relevant to explore its possible immunomodulatory effects. In particular, compounds such as thymoquinone, alpha-herein, carvacrol, beta-sitosterol, isoquinoline alkaloids, and various fatty acids found in *N. sativa* have shown the ability to influence immune responses, either through regulation of cytokines, activation of immune cells or indirect support to the immune system cell function. This dual approach, which simultaneously targets neuropilins and enhances the immune system’s ability to fight breast cancer cells, holds promise for the development of more comprehensive and effective therapeutic strategies for breast cancer patients, warranting deeper investigation.

In siliconsilicon analysis, we investigated the binding free energy (ΔG) of Neuropilins, an anti-cancer therapy targeting angiogenesis through the inhibition of vascular endothelial growth factor (VEGF), with various signaling pathways including CDK4, EGFR, RAS, BRAF, PI3K, and PTEN. Our *in silico* investigation revealed that hydrogen bonding is pivotal in inducing conformational changes within the DNA structure, impeding its replication and preventing cell death. Molecular docking results revealed the presence of crucial hydrogen bonds and supported the formation of stable Neuropilins complexes. The molecular docking scores for the tested complexes were CDK4 (Score = −7.2 kcal/mol), EGFR (Score = −8.5 kcal/mol), RAS (Score = −6.9 kcal/mol), BRAF (Score = −7.8 kcal/mol), PTEN (Score = −6.5 kcal/mol), and PI3K (Score = −8.3 kcal/mol). The binding mode demonstrated vital hydrogen bonds correlated with the observed energy gap. Notably, the calculated binding free energies of the tested compounds are as follows: CDK4 (ΔG = 24275.195 ± 6411.293 kJ/mol), EGFR (ΔG = 363273.625 ± 8731.466 kJ/mol), RAS (ΔG = 181751.990 ± 28438.515 kJ/mol), BRAF (ΔG = 162414.725 ± 10728.367 kJ/mol), PTEN (ΔG = 40162.585 ± 4331.017 kJ/mol), and PI3K (ΔG = 434783.463 ± 53989.676 kJ/mol). Furthermore, through extensive 200 ns MD simulations, we observed the formation of a stable Neuropilins complex structure. The simulations confirmed the stability of the Neuropilins complex with the signaling pathways.

In silico studies have limitations and need validation through extensive *in vitro* and *in vivo* experiments (Viceconti et al., 2021). Breast cancer is not the same in all patients as we perceived results by Campos et al. (2022), so NRP-targeted therapy may have different effects depending on the subtype of breast cancer. If the findings are supported by strong *in-vitro* data, NRP-targeted therapies could progress to clinical trials. However, there are still obstacles to overcome, like potential side effects and figuring out the best way to use NRP-targeted therapy in patients. In this study, we present the *in silico* and *in vitro* studies exploring NRPs as targets for multi-coded oncogenes in breast cancer show great promise. Integrating computer-based predictions with real-life experiments helps identify new treatment strategies. However, more research is needed to fully understand the potential of NRP-targeted therapies for breast cancer patients. The study’s findings could contribute to the development of personalized medicine, reduce side effects, enhance our scientific understanding, and potentially improve the lives and outcomes of breast cancer patients, fostering hope and economic benefits in the process.

## 5 Conclusion

In our investigation of *N. sativa* leaves, our phytochemical analysis unveiled the presence of tannins, saponins, steroids, and cardiac glycosides, while phlorotannins and terpenoids were notably absent. The quantitative estimation revealed a substantial alkaloid content of 9.4% ± 0.04% and saponins at 1.9% ± 0.05%. Among the various extracts, methanol extract emerged as the most prolific, boasting the highest yield (4). Furthermore, it exhibited the most robust antioxidant profile, with Total Flavonoid Content at 127.51 ± 0.76 mg/100 g and Total Phenolic Content at 134.39 ± 0.589 mg GAE/100 g. Notably, our Total Antioxidant Capacity (TAC) assay demonstrated a concentration-dependent antioxidant effect, underlining the potential health benefits of *N. sativa* leaves. Moreover, our hemolytic activity testing uncovered varying degrees of hemolysis (ranging from 3.7% to 16.5%), suggesting the need for careful consideration of extraction methods and dosage in potential applications. In the realm of virtual experimentation, our *in silico* studies shed light on the promising potential of Neuropilins and their derivatives as effective anticancer agents, with a particular affinity for targeting the PI3K protein and its associated pathways. These findings hold considerable promise for the development of targeted therapies in the fight against cancer. Our study underscores the remarkable medicinal potential of *N. sativa* leaves, driven by their rich bioactive compounds and antioxidant properties. Furthermore, our exploration of Neuropilins as prospective anticancer agents opens exciting avenues for future research and development in the field of cancer therapeutics. Subsequent investigations are warranted to elucidate and harness the full extent of their capabilities as a potent weapon against cancer.

## Data Availability

The raw data supporting the conclusions of this article will be made available by the authors, without undue reservation.
